# Hypomethylating agents induce epigenetic and transcriptional heterogeneity with implications for acute myeloid leukemia cell self-renewal

**DOI:** 10.1038/s41375-025-02693-5

**Published:** 2025-07-17

**Authors:** Danielle R. Bond, Sean M. Burnard, Kumar Uddipto, Kooper V. Hunt, Brooke M. Harvey, Luiza Steffens Reinhardt, Charley Lawlor-O’Neill, Ellise A. Roper, Sam Humphries, Heather C. Murray, Abdul Mannan, Matthew D. Dun, Charles E. de Bock, Nikola A. Bowden, Anoop K. Enjeti, Nicole M. Verrills, Carlos Riveros, Kim-Anh Lê Cao, Heather J. Lee

**Affiliations:** 1 School of Biomedical Science and Pharmacy, The University of Newcastle, Callaghan, NSW Australia; 2 Precision Medicine Research Program, Hunter Medical Research Institute, New Lambton Heights, NSW Australia; 3 Cancer Detection and Therapy Research Program, Hunter Medical Research Institute, New Lambton Heights, NSW Australia; 4 New South Wales Health Pathology, John Hunter Hospital, New Lambton Heights, NSW Australia; 5 Children’s Cancer Institute, Lowy Cancer Research Centre, Randwick, NSW Australia; 6 School of Clinical Medicine, Faculty of Medicine, UNSW, Randwick, NSW Australia; 7 School of Medicine and Public Health, The University of Newcastle, Callaghan, NSW Australia; 8 Drug Repurposing and Medicines Program, Hunter Medical Research Institute, New Lambton Heights, NSW Australia; 9 Department of Haematology, Calvary Mater Hospital, Waratah, NSW Australia; 10 Melbourne Integrative Genomics, School of Mathematics and Statistics, The University of Melbourne, Parkville, VIC Australia

**Keywords:** Cancer therapeutic resistance, Acute myeloid leukaemia, Cancer epigenetics

**To The Editor:**

DNA hypomethylating agents (HMAs), such as azacytidine (AZA, 5-azacytidine) and decitabine (DAC, 2’-deoxy-5-azacytidine), are used to treat acute myeloid leukaemia (AML) and myelodysplastic neoplasm. However, low response rates and therapy-resistant relapse remain significant challenges [1]. Reasoning that HMA-resistant relapse could originate from rare cells that evade and adapt to treatment, we sought to characterise heterogeneous responses to HMA treatment in AML cells.

AZA and DAC are cytidine analogues, which are incorporated into DNA during replication [1]. This leads to the degradation of DNA methyltransferase (DNMT) enzymes, loss of DNA methylation in subsequent cell divisions, and pleiotropic transcriptional changes. Unlike DAC, AZA can also be incorporated into RNA, which influences transcript stability and translation [2]. HMAs also influence pyrimidine metabolism [3].

In this study, AML cell lines (HL-60, MOLM-13, and MV-4-11; Supplementary Table 1) were stained with CellTrace, and treated with low-dose DAC or AZA (Supplementary Fig. S1) in suspension culture (Fig. 1A). After 72 h (experiment day 3), cells were collected for single-cell analyses (Fig. 1), or seeded into MethoCult media for colony-forming assays. After an additional 14 days (experiment day 17), individual colonies were picked for molecular analyses (Fig. 2). Detailed methods are included in the Supplementary Material.

**Fig. 1:**
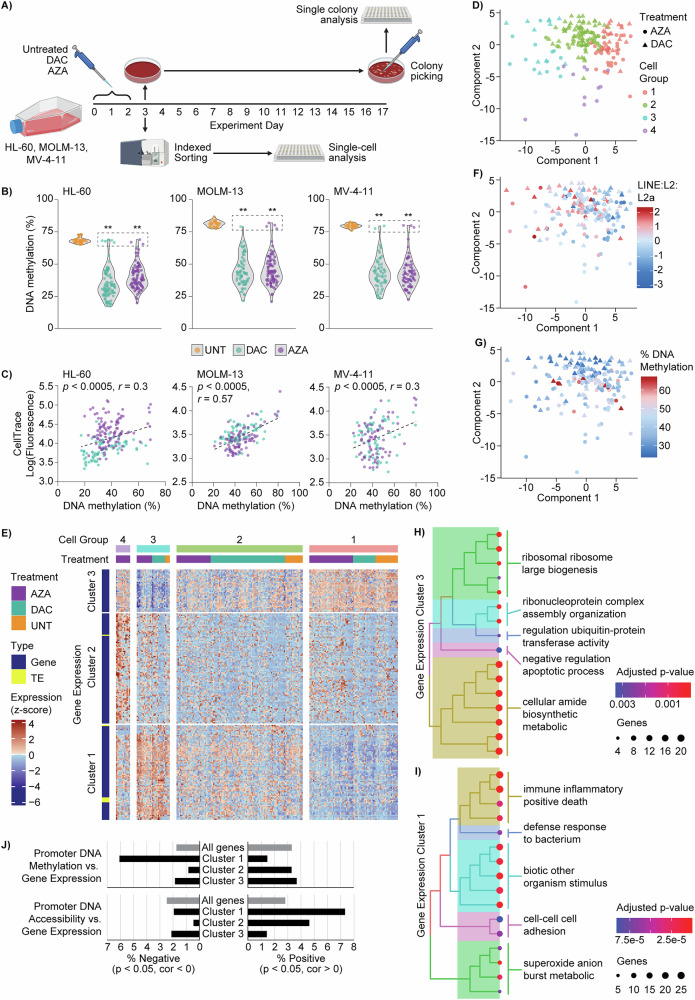
HMA treatment induces DNA methylation heterogeneity in AML cells. **A** Schematic of experiment design. HL-60, MOLM-13 and MV-4–11 cells were labelled with CellTrace and treated with decitabine (DAC; 100 nM) or azacytidine (AZA; HL-60: 2000 nM, MOLM-13 and MV-4-11: 500 nM) every 24 h for 72 h. Single cells collected by indexed FACS on experiment day 3 were subjected to scNMT-seq (HL-60) or scTEM-seq (MOLM-13, MV-4-11). Created in BioRender. Lee, H. (2025) https://BioRender.com/1y2phwx**B** Violin plots of DNA methylation levels in single HL-60 (left), MOLM-13 (middle) and MV-4-11 (right) cells. Superimposed points show single-cell values from untreated (UNT, orange), DAC (cyan) and AZA (purple) groups. Dashed boxes surround DAC and AZA cells with methylation levels within the range of UNT samples. Data are shown for 185-222 cells from 2–3 replicate experiments in each cell line (UNT, *n* = *27-38*; DAC *n* = *63-93*; AZA *n* = *68-91*). Statistical analysis was performed using ordinary one-way ANOVA with Dunnett’s (multiple comparisons test: ** *p* < 0.0005 vs. UNT). **C** Scatter plots comparing CellTrace fluorescence and DNA methylation in single cells, with linear regressions, F-test *p*-values, and Pearson correlation coefficients (*r*). **D** sPLS projection of HMA treated (AZA and DAC only) HL-60 cells based on transcript features coloured by cell group 1–4 (from E). **E** Heatmap of transcript features selected by sPLS displaying all samples (treated and untreated) as columns, are split by k-means clustering and grouped by treatment. Individual gene and TE expression levels (rows) are z-score normalised and split by k-means clustering with internal hierarchical clustering. **F** and **G** sPLS projections coloured by **F** LINE:L2:L2a expression, and **G** global methylation level. **H** and **I** Simplified tree plots of the gene ontology analysis for gene expression clusters 3 and 1. **J** Pearson correlations were computed between gene expression and DNA methylation (left) or accessibility (right) of associated promoters. Bar graphs show the percentage of correlations (*p* < 0.05) with negative and positive coefficients for all genes and filtered by gene expression cluster (identified in E).

**Fig. 2:**
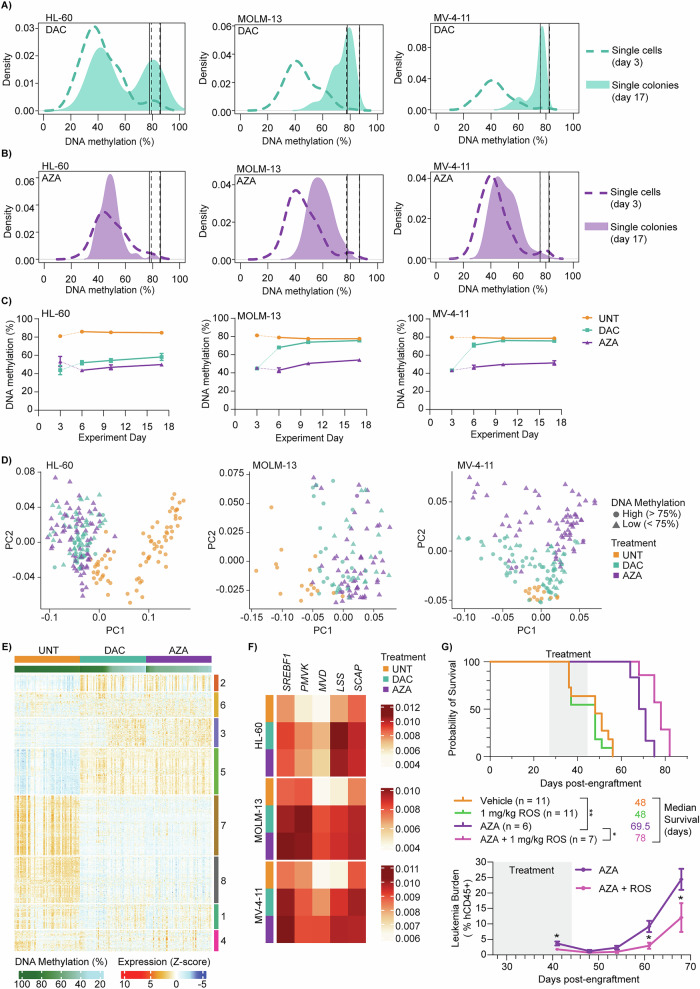
HMA-induced heterogeneity has implications for AML cell self-renewal and co-treatment strategies. AML cell lines were treated with DAC or AZA in suspension culture as described in Fig. 1. On experiment day 3, cells were seeded in MethoCult media for colony formation, without additional HMA treatment. On experiment day 17, colonies were counted and individual colonies were manually picked for sequencing analysis. **A** and **B** Density plots show the average DNA methylation levels for single cells collected on experiment day 3 (dashed line, from Fig. 1B) and individual colonies collected on experiment day 17 (solid fill) following treatment with DAC or AZA. Vertical lines indicate the minimum and maximum values for untreated single cells (dashed) and colonies (solid). HL-60 scNMT-seq data were filtered for cytosines within SINE Alu sites for direct comparison to scTEM-seq data from colonies. Data are shown for 288 colonies collected from triplicate experiments in each cell line (*n* = *96* per treatment). **C** Time-course experiment showing changes in average DNA methylation of cells collected at different time points throughout the colony-forming assay (experiment days 6, 10 and 17). Values for experiment day 3 were obtained from single-cell data (Fig. 1B). Data are expressed as mean +/- standard error of the mean (SEM). **D** Principal Component Analysis (PCA) plots of single-colony RNA-seq data from AML cell lines, highlighting treatment groups (UNT = orange; DAC = cyan; AZA = purple) and matched mean DNA methylation levels (circle: high >75%; triangle: low <75%). Data shown for 119–220 colonies collected from three replicate experiments in each cell line (UNT, *n* = *14-73*; DAC, *n* = *46-78*; AZA, *n* = *56-73*). **E** Heatmap of the top 2000 highly variable genes from HL-60 colony RNA-seq data. Samples are ordered by decreasing global methylation levels (green gradient) within each treatment group. Rows are grouped by K-means clusters based on gene expression, with hierarchical clustering by Euclidean distance within each cluster. **F** Heatmap of five genes (SREBF1, PMVK, MVD, LSS and SCAP) from the cholesterol biosynthesis pathway (GO:0006695), from colony RNA-seq data in all three cell lines. Values displayed are the mean expression of samples within each treatment group. **G** Survival analysis (top) and leukaemia burden (% human CD45^+^ cells, bottom) for the AML-16 patient-derived xenograft model following treatment with AZA (1 mg/kg/day) +/- rosuvastatin (1 mg/kg/day) for 5 days on, 2 days off in cycle 1, followed by twice per week (dispersed) for an additional two cycles, via intraperitoneal (IP) injection. Survival analysis was performed using Kaplan-Meier analysis followed by the Log-rank (Mantel-Cox) test and a *p*-value of <0.05 was considered statistically significant. Analysis of leukaemia burden was performed using the Mann-Whitney test (unpaired, non-parametric, two-tailed t-test) with a *p*-value cut-off of 0.05. *p* < 0.05*, *p* < 0.005**.

Single-cell analysis revealed striking intercellular DNA methylation heterogeneity after HMA treatment (Fig. 1B; Supplementary Table 2), with the extent of hypomethylation varying substantially among cells treated with DAC (e.g., HL-60: 17–69%) or AZA (e.g., HL-60: 20–69%). This was related to cell division (Fig. 1C; Supplementary Fig. S2), consistent with HMA incorporation during replication. Interestingly, a small proportion (1–5%) of methylation-retaining cells (Fig. 1B, dashed boxes) displayed no evidence of HMA-induced hypomethylation, with DNA methylation of at least the minimum observed in untreated cells.

To explore the effects of DNA methylation heterogeneity on other layers of genetic regulation, we used multi-omic scNMT-seq data collected from HL-60 cells. HMA-induced DNA methylation heterogeneity was observed in almost all genomic contexts, and was associated with significantly weakened correlations to DNA accessibility and gene expression across the genome (Supplementary Fig. S3).

Variably expressed transcripts (genes and transposable elements) with correlations to DNA methylation and chromatin accessibility were then identified using an unsupervised sparse Partial Least Squares (sPLS) method. This identified 4 groups of cells with different patterns of expression across three gene clusters (Fig. 1D, E; Supplementary Table 3). A significant and negative correlation (*r* = −0.37, *p* < 1 × 10^-7^) between TE LINE:L2a expression and component 1 was observed (Fig. 1F; Supplementary Fig. S4), consistent with HMA-induced activation of viral mimicry [4] in cell group 3. However, there was no significant relationship between global DNA methylation and component 1, with all cell groups having similar methylation levels (Fig. 1G; Supplementary Fig. S4).

Cell group 3 had low expression of genes in cluster 3 that were related to translation and inhibition of cell death (Fig. 1H; Supplementary Table 4). Cell group 3 also had high expression of genes in cluster 1 (Fig. 1I) that were enriched in terms related to immune inflammatory response and positive regulation of cell death. This transcriptional profile is consistent with the expected effects of HMA treatment [1], and 29 of the 78 genes in cluster 1 were significantly upregulated by DAC and/or AZA in matched bulk RNA sequencing (RNA-seq) data (Supplementary Table 3). Cell group 1 displayed an inverted gene expression pattern when compared to cell group 3 (high expression of cluster 3 and low expression of cluster 1 genes). Untreated cells were also over-represented in cell group 1 (observed: expected ratio = 1.48). This suggests that cell group 1 did not activate transcriptional pathways commonly associated with HMA treatment, despite low methylation levels in most cells.

The sPLS model also selected epigenetic features that were correlated to variably expressed transcripts (Supplementary Tables 5, 6). Genes from expression cluster 1 had predominantly positive correlations with accessibility features and many negative correlations with methylation features, especially in CpG islands and 3 kb genomic windows (Supplementary Fig. S5). To test whether epigenetic alterations in *cis*-regulatory elements could influence transcriptional responses to HMA treatment, we also correlated gene expression with DNA methylation and accessibility in nearby loci. Genes from expression cluster 1 showed a significant shift toward negative correlations with promoter methylation (*p* = 2.2 × 10^−11^, χ^2^ test) and positive correlations with promoter accessibility (*p* = 2.3 × 10^−8^, χ^2^ test), suggesting that these genes are particularly sensitive to loss of DNA methylation in *cis* (Fig. 1J; Supplementary Table 7).

Next, we asked whether methylation-retaining cells have a relative growth advantage following HMA treatment using colony-forming assays and single-colony sequencing (Supplementary Fig. S6). Many colonies derived after DAC treatment had DNA methylation levels ≥75% at experiment day 17 (Fig. 2A, solid fill; Supplementary Table 8). This was in stark contrast to the low percentage of methylation-retaining cells previously seen in single-cell data at day 3 (Fig. 1B; Fig. 2A, dashed line). Interestingly, increased DNA methylation was observed just 3 days after DAC withdrawal in MOLM-13 and MV-4-11 colonies (Fig. 2C), suggesting that methylation-retaining cells could be more likely to form colonies than hypomethylated cells. Meanwhile, very few colonies established after AZA treatment had DNA methylation levels ≥75% (Fig. 2B), and these samples showed no increase in methylation during colony formation (Fig. 2C). DNA methylation was also low for more than 1 week after HMA withdrawal in a suspension culture experiment (Supplementary Fig. S1E). Thus, rapid recovery of DNA methylation is unlikely to explain the prevalence of highly-methylated colonies observed after DAC treatment. Rather, we propose that methylation-retaining cells can have increased self-renewal and proliferative capacity relative to hypomethylated cells.

Single-colony RNA-seq showed that DAC and AZA samples were generally concordant and distinct from untreated samples, regardless of global DNA methylation levels (Fig. 2D; Supplementary Table 8). This implies that HMA exposure has substantial effects on the transcriptome, even in highly methylated cells. Of the 2000 most variably expressed genes among the HL-60 samples, only 215 had increased expression specific to hypomethylated colonies (Fig. 2E, cluster 3; Supplementary Table 9). Many of these genes (42.3%) were upregulated after 72 h of treatment with either DAC or AZA in bulk HL-60 RNA-seq data, and several were associated with activation of inflammatory responses within the sPLS model (e.g., *S100A8* and *S100A9*; Supplementary Table 9; Fig. 1E, cluster 1). In contrast, the genes in cluster 5 were up-regulated following HMA exposure in both hypomethylated and highly-methylated colonies (Fig. 2E). Gene ontology analysis revealed an enrichment of anti-microbial and immune-related processes among both hypomethylation-dependent and -independent gene sets, whereas ‘small molecule biosynthetic process’ and ‘cholesterol biosynthetic process’ were over-represented among the hypomethylation-independent cluster 5 genes (Supplementary Fig. S7; Supplementary Table S10). HL-60 colonies that retained DNA methylation following DAC treatment also had particularly high expression of cholesterol-related genes, including many enzymes required for de novo cholesterol biosynthesis downstream of mevalonate [5] (Supplementary Fig. S8–9; Tables S11, S12).

In a focused analysis of ‘cholesterol biosynthetic process’ (GO:0006695) colonies displayed up-regulation of many genes after HMA treatment (Supplementary Fig. S10), and five genes (*SREBF1*, *SCAP*, *PMVK*, *MVD,* and *LSS*) were significantly increased by both DAC and AZA in all cell lines (Fig. 2F, Supplementary Fig. S11). These genes form part of a regulatory feedback loop that increases cholesterol biosynthesis when intracellular cholesterol levels are low (Supplementary Fig. S9). Since cholesterol regulation is altered in both methylation-retaining and hypomethylated colonies, targeting this pathway could overcome the initial heterogeneity induced by HMA treatment (Fig. 1).

We next performed colony-forming assays in the presence of rosuvastatin, a potent inhibitor of the rate-limiting enzyme of the cholesterol biosynthetic pathway (HMGCR). AZA and rosuvastatin co-treatment had synergistic effects to inhibit colony formation in MV-4-11 cells, while DAC and rosuvastatin had synergistic effects in all cell lines (Supplementary Fig. S12).

The efficacy of DAC and rosuvastatin co-treatment was also tested in vivo. In MOLM-13 xenografts, co-treatment significantly increased median survival compared to DAC alone (Supplementary Fig. S13; Supplementary Table 13, 14). Similarly in a patient-derived xenograft model, rosuvastatin co-treatment significantly extended the median survival of AZA-treated mice (Fig. 2G; Supplementary Table 15) and decreased leukaemia burden (Supplementary Fig. S13).

While several studies have explored heterogeneous responses to HMA treatment [6–10], our unique study is the first to reveal HMA-induced DNA methylation heterogeneity (Fig. 1B) with implications for transcriptional programmes (Fig. 1D–J) and AML cell self-renewal (Fig. 2A–C). We show that heterogeneous loss of DNA methylation is related to cell division (Fig. 1C), but many other factors are also likely to contribute. For example, differences in cell cycle, cell maturation, drug uptake and metabolism, DNMT1 depletion, and activation of apoptotic and/or differentiation pathways, could all influence a cell’s response to HMA treatment. We also demonstrate that increased cholesterol biosynthesis facilitates the self-renewal of AML cells following HMA exposure (Fig. 2D–F), and that rosuvastatin co-treatment enhances HMA effects in vitro and in vivo (Fig. 2G). These findings add to a growing interest in cholesterol regulation in HMA-treatment of AML and myelodysplastic neoplasm [11–15] and support further investigation of the long-term benefits of statin and HMA co-treatments.

## Supplementary information

Supplementary Material

Supplementary Tables

## Data Availability

The datasets generated and analysed in the current study are available in the GEO database (GSE256354). Relevant code for this manuscript is available on GitHub via: www.github.com/canepi/HMA_heterogeneity.
