# Supplementary material for: Hypomethylating agents induce epigenetic and transcriptional heterogeneity with implications for acute myeloid leukemia cell self-renewal
Source: Leukemia. 2025 Jul 17;39(9):2275–80. doi: 10.1038/s41375-025-02693-5 (PMC12380606; doi:10.1038/s41375-025-02693-5)
Supplement: Supplementary file 1 — Supplementary Material [file 41375_2025_2693_MOESM1_ESM.docx]

# Hypomethylating agents induce epigenetic and transcriptional heterogeneity with implications for acute myeloid leukemia cell self-renewal

Danielle R Bond^1,2^*^, Sean M Burnard^1,2^^, Kumar Uddipto^1,2^, Kooper V Hunt^1,2^, Brooke M Harvey^1,2^, Luiza Steffens Reinhardt^1,3,4^, Charley Lawlor-O’Neill^1^, Ellise A Roper^1^, Sam Humphries^1,2^, Heather C. Murray^1,2^, Abdul Mannan^1,2^, Matthew D Dun^1,2^, Charles E de Bock^5,6^, Nikola A Bowden^7,8^, Anoop K Enjeti^2,4,7,9^, Nicole M Verrills^1,2^, Carlos Riveros^7^, Kim-Anh Lê Cao^10^, Heather J Lee^1,2*^

*^1^School of Biomedical Science and Pharmacy, The University of Newcastle, Australia.*

*^2^Precision Medicine Research Program, Hunter Medical Research Institute, Australia.*

*^3^Cancer Detection and Therapy Research Program, Hunter Medical Research Institute, Australia.*

*^4^New South Wales Health Pathology, John Hunter Hospital, Australia.*

*^5^Children’s Cancer Institute, Lowy Cancer Research Centre, Randwick, NSW 2031, Australia*

*^6^School of Clinical Medicine, Faculty of Medicine, UNSW Sydney, NSW 2052, Australia*

*^7^School of Medicine and Public Health, The University of Newcastle, Australia.*

*^8^Drug Repurposing and Medicines Program, Hunter Medical Research Institute, Australia.*

*^9^Department of Haematology, Calvary Mater Hospital, Australia.*

*^10^Melbourne Integrative Genomics, School of Mathematics and Statistics, The University of Melbourne, Australia.*

^ These authors contributed equally to this work; * Corresponding authors

# Supplementary Information

## Materials and Methods

## Supplementary References

## Supplementary Figures S1 to S13

## Materials and Methods

### Cell lines and culture

AML cell lines (Supplementary Table 1), HL-60 (ATCC #CCL-240), MOLM-13 (DSMZ #ACC-554), and MV-4-11 (ATCC #CRL-9591), were maintained in tissue culture flasks (Greiner Bio-One) at 37°C and 5% CO_2_, and sub-cultured at 500,000 cells/mL every 2-3 days with fresh medium. HL-60 cells were maintained in Iscove′s Modified Dulbecco′s medium (IMDM; Sigma-Aldrich) supplemented with 4 mM GlutaMAX (Thermo Fisher Scientific) and 10% Fetal Bovine Serum (FBS; Sigma-Aldrich). MOLM-13 and MV-4-11 cells were maintained in Roswell Park Memorial Institute 1640 medium (RPMI; Sigma-Aldrich) supplemented with 2 mM GlutaMAX and 10% FBS.

### CellTrace staining

AML cells (2-2.5x10^6^ cells/mL) were stained with 1 µM (MOLM-13 and MV-4-11) or 3 µM (HL-60) CellTrace Far Red (Thermo Fisher Scientific), according to the manufacturer’s instructions. MOLM-13 and MV-4-11 cells with uniformly high CellTrace fluorescence were purified by fluorescence-activated cell sorting (FACS) prior to treatment with hypomethylating agents (HMAs), whereas all cells were used for HL-60 treatments.

### HMA treatments

HMA treatments were performed in suspension culture, with drugs added every 24 h for 72 h total. All cell lines were treated with 100nM decitabine (DAC; Selleckchem #S1200), HL-60 cells were treated with 2000nM azacytidine (AZA; Selleckchem # S1782), while MOLM-13 and MV-4-11 cells were treated with 500nM AZA. Untreated cells (UNT) received 0.1% DMSO (vehicle control).

### Fluorescence activated cell sorting (FACS)

HMA-treated cells were stained with propidium iodide (PI, 1.5 µg/mL) and prepared for FACS. Viable (PI^-^) single cells were sorted into 2.5 µL of RLT PLUS buffer (Qiagen) containing 2.5 U SUPERas-In (Thermo Fisher Scientific) in 96-well plates using indexed sorting on a FACS Aria II (BD Biosciences). Plates were sealed and briefly centrifuged before storage at -80°C for sequencing analysis.

### Colony-forming assays

HMA-treated cells were seeded in MethoCult Optimum (H4034; STEMCELL Technologies Inc.) at 500 cells/well in 6-well plates with rosuvastatin (Selleckchem # S2169) added to the MethoCult at various doses (0, 1, 10, 30 µM). Cells were cultured at 37°C and 5% CO_2_ for 14 days, and colonies were imaged using Cytation3 (Biotek). Colony counts and sizes were analyzed using ImageJ software. Individual colonies were manually plucked using a 20 µL pipette tip into 100 µL of media, centrifuged at 200xg for 5 min, and then resuspended in 20 µL of RLT PLUS buffer before storage at -80°C. Alternatively, all colonies in each well were collected by resuspending the MethoCult Optimum media (and colonies) in 3 mL of standard culture media (IMDM or RPMI), centrifuging at 200xg for 5 min, and resuspending the cell pellet in 20-50 µL of RLT PLUS buffer before storage at -80°C.

### Single-cell sequencing analyses

#### scNMT-seq library preparation and sequencing

For scNMT-seq, matched scNOMe-seq and scRNA-seq libraries were prepared from sorted HL-60 single cells as previously described (1). Minor modifications to the published protocol were as follows: 1) Amplified cDNA was purified using a 0.6:1 volumetric ratio of AMPure XP Beads (Beckman Coulter) and eluted in 15 µL water. 2) During the fifth repeat of first-strand synthesis reaction, the samples were held at 37°C for 90 min. 3) The oligo used in the second-strand synthesis reaction (5’-CAGACGTGTGCTCTTCCGATCTNNNNNN-3’) was modified to be compatible with the NEBNext oligos (multiplex oligos for Illumina, dual index sets, New England Biolabs), which were used to amplify scNOMe-seq libraries. 4) A 0.65:1 volumetric ratio of AMPure XP Beads was used to purify products of both the first- and second-strand synthesis reactions, as well as the amplified scNOMe-seq libraries.

For scNOMe-seq libraries, paired-end 150 bp sequencing was performed using the NovaSeq (Illumina) platform. For scRNA-seq libraries, paired-end 75 bp sequencing was performed on the NovaSeq or NextSeq (Illumina) platform.

#### scTEM-seq library preparation and sequencing

For scTEM-seq analysis of global DNA methylation levels in single MOLM-13 and MV-4-11 cells, library preparation was performed as described (2). Paired-end 150 bp sequencing was performed on the MiSeq (Illumina) platform.

#### scNOMe-seq data processing

Single-cell bisulfite libraries were processed using Bismark (3) (v0.22.3) as described (1). Following demultiplexing, samples with multiple sequencing lanes per read were concatenated into single R1 and R2 fastq files per sample. Reads were trimmed using Trim Galore (4) (v0.6.6) with cutadapt (v1.18) to remove automatically detected illumina adaptors and the first 9 reads ‘--Clip_R1 9’ in single end mode. Reads were then mapped by single-end non-directional alignment to Bowtie2 indexed human genome GRCh38. Sample reads were deduplicated in single-end mode and concatenated into a single file per sample by ‘deduplicate_bismark --multiple --single’. Methylation extraction and coverage file generation was performed separately on samples with ‘bismark_methylation_extractor --single-end --bedGraph --gzip --cx’, followed by ‘coverage2cytosine --gzip --nome --seq’. For analyses comparing HL-60 scNOMe-seq data and colony TEM-seq data (Fig. 2A, B), scNOMe-seq data was filtered for CpG sites overlapping SINE Alu sites.

#### scTEM-seq data processing

Bisulfite sequencing data processing and extraction of global methylation estimates for single-cell samples were performed as previously described (2, 5) and scripts available via <https://github.com/canepi/scTEM-seq>. In scTEM-seq data from MOLM-13 and MV-4-11 cells, samples were excluded if fewer than 500 unique SINE Alu annotations were represented.

#### scRNA-seq data processing

Following demultiplexing, multiple sequencing lanes per read were concatenated into single R1 and R2 fastq files, per sample. Raw reads were trimmed in pair-end mode using Trim Galore (v0.6.5) and Cutadapt (v2.10), retaining unpaired reads. Hisat2 (6) (v2.1.0) was used to map and align trimmed and unpaired reads using default parameters (--phred33) to the human reference genome build (GRCh38), and samtools (v1.10) was used to sort by coordinate and generate bam files.

TEtranscripts (7) (v2.2.1) was used to obtain raw gene and transposable element counts from unique and ambiguously aligned reads (bam files) using TEcount with the flags '--sortByPos --mode multi’. The GTF files used were generated as follows: 1) TEs (<http://hgdownload.soe.ucsc.edu/goldenPath/hg38/database/rmsk.txt.gz>; USCS repeating element Repeat Masker; hg38; 2021-09-03) and 2) genes (<ftp://ftp.ebi.ac.uk/pub/databases/gencode/Gencode_human/release_30/gencode.v30.annotation.gtf.gz>; gencode Release 30; GRCh38.p12; 2019-04-08). To ensure the GTF files worked with
TEtranscripts the gene GTF (from gencode) was updated by converting ‘M’ to ‘MT’ for mitochondrial genes in the chromosome column and removal of ‘Chr’ preceding each chromosome number. The TE GTF was generated from the repeat mask file using the conversion tool from the TEtranscripts developers (<http://labshare.cshl.edu/shares/mhammelllab/www-data/TEtranscripts/TE_GTF/makeTEgtf.pl.gz>) ‘perl makeTEgtf.pl -c 6 -s 7 -e 8 -o 10 -t 11 -n hg38_rmsk -f 13 -C 12 -S 2 rmsk.txt >> hg38_rmsk_TE.gtf’ and removing ‘chr’ from chromosome names ‘sed 's/chr//' hg38_rmsk_TE.gtf > hg38_rmsk_TE_NoChr.gtf’.

#### scNMT-seq quality control

For scNMT-seq data, cells were required to pass both scNOMe-seq and scRNA-seq quality control (QC). Cells that had less than 500,000 CpG sites covered, less than 5,000,000 GpC sites covered, greater than 15% CHH methylation rate, or less than 2% GpC methylation failed scNOMe-seq QC. For scRNA-seq, QC was performed using bam files from hisat2 and the SeqMonk (v1.47.1) ‘RNA-seq QC Plot’. Cells that had less than 70% reads in exons or less than 15% genes measured failed scRNA-seq QC. In total, 222 scNMT-seq samples passed QC (Table S2).

#### scNOMe-seq normalisation and batch correction

scNOMe-seq libraries provide information on both DNA methylation (CpG sites) and DNA accessibility (GpC sites). For both CpG (methylation) and GpC (accessibility) datasets, several genomic annotation contexts were considered, including introns, exons, intergenic regions, CpG islands, promoters (-1500 bp to +500 bp of transcription start sites), H3K4me3 sites (ENCODE (8) accession ID: ENCFF021JBH, experiment: ENCSR000DUO), and H3K27ac sites (ENCODE (8) accession ID: ENCFF763UAG, experiment: ENCSR919WLM). In addition, unbiased 3 kb windows of the whole genome were generated with a step size of 1.5 kb.

For DNA methylation, the CpG methylation rate was estimated within each annotation window using the Bayes binomial approximation, as in Smallwood *et. al.* (9).

The GpC methylation, which marks accessible DNA in scNOMe-seq libraries, was introduced *in vitro* using a bacterial GpC methyltransferase enzyme. To remove batch effects resulting from differences in enzymatic activity, data normalization and batch correction were performed as follows. GpC data for the whole genome were aggregated in windows of 500 kb in length with 250 kb overlap separately for methylated and unmethylated GpC counts for each cell. Per-cell pooled size factors were computed from these 500 kb windows using the method of Lun *et. al.* (10) scaling by total library size, as implemented in the single-cell R package scuttle (v1.8.4) (11). Batch scaling factors were estimated from corrected methylated and unmethylated window log counts using the rescaleBatches method from the R package batchelor (v1.14.1) (12). Per cell methylated and unmethylated cell-scaling factors were calculated as the ratio of the batch-corrected sum of counts to the mean sum of counts across cells. Finally, unscaled methylated and unmethylated counts in each cell were independently scaled by the product of the cell pooled size factor and methylated/unmethylated count batch correction factor, respectively. The GpC methylation rate for each annotation window was then computed using normalized batch-corrected counts by Bayesian binomial approximation.

From the overall distribution of counts across the annotation layer for CpG and GpC methylation data, minimum total count thresholds per window of 5 counts (CpG) and 20 counts (GpC) were established and applied to discard windows with unreliable methylation rate estimations.

#### scRNA-seq normalisation and batch correction

scRNA-seq libraries from HL-60 scNMT-seq data were filtered to remove lowly expressed genes, requiring at least five counts in 10% of the cells. Normalization and variance stabilization were performed by the scTransform method (13) and batch correction by anchor-based integration using the R package Seurat (v4.2.0) (14). First, the batches were independently normalized using scTransform. Then, the top 5,000 most variable features that were in common across batches were identified and used to determine integration anchors. These features and anchors were then used for integration, thereby retaining those 5,000 commonly variable features. Finally, a sparse RNA-seq matrix was utilized for some analyses, whereby gene imputation calculations, performed by Seurat during integration, were ignored and removed by reintroducing ‘NAs’ in place of genes with originally ‘missing data’ (zeros). All downstream analyses only considered autosomal genes (Chr1-22).

#### Pairwise dissimilarity analysis of DNA methylation heterogeneity

To assess DNA methylation heterogeneity per treatment group and genomic context (Supplementary Fig. S3B), pairwise CpG methylation dissimilarity analysis was performed. The mean absolute methylation difference was computed for each cell pair (A, B) as the mean of the absolute difference in methylation rate at each common cytosine position in the relevant genomic annotation. To make the comparison of methylation patterns meaningful, only cytosine loci with data from both cells in the pair were used. The mean absolute methylation differences were grouped according to the treatment combination of the cell pairs. The global summaries shown in Supplementary Fig. S3B correspond to groups in which both cells in the pair had the same treatment. Higher values indicate a more heterogeneous methylation pattern when cells in the same treatment group were compared vis-à-vis.

#### Cell-wise correlation analysis

To assess the relationships between DNA methylation, DNA accessibility, and gene expression within individual cells (Supplementary Fig. S3C), Pearson correlations were computed using HL-60 scNMT-seq data. For this analysis, RNA-seq data was normalized and log transformed per batch using ‘scuttle::logNormCounts()’ (v1.6.2) (11) without batch correction or prior count filtering. DNA methylation was correlated to DNA accessibility at matched loci, based on genomic co-ordinates. For correlations with gene expression, methylation and accessibility measurements at promoters, introns, and exons were matched to the corresponding transcript. For CpG islands, H3K27ac sites and H3K4me3 sites, methylation and accessibility measurements were matched to all transcripts within 10 kb. For each cell, Pearson’s correlation estimates were then computed using all matched values and the ’cor.test()’ function in R.

#### Integrative sparse partial least squares (sPLS) analysis

Mixomics (15) (v6.20.0) was used to perform a multivariate integrative analysis of the HL-60 scNMT-seq data (Fig. 1D-H). Feature selection was performed to identify variably expressed transcripts that were highly correlated with changes in DNA Methylation and accessibility after HMA treatment. We performed an unsupervised sparse Partial Least Squares (sPLS) analysis using the function ‘mixOmics::mint.block.spls()’ which combines a multivariate integrative (MINT) method and a multiblock sPLS integrative analysis. MINT (16) accounts for multiple batches (Supplementary Table 2) measured on the same variables, while the multiblock sPLS seeks for correlated patterns between DNA methylation and DNA accessibility rates that are split into multiple genomic regions (‘blocks’) and explain (correlated to) the predictor (transcriptome).

To focus on transcriptomic and epigenetic changes resulting from HMA treatment, only treated cells (AZA and DAC) were included in the sPLS model. The genomic regions included in this analysis for both DNA methylation and DNA accessibility were CpG islands, promoters, H3K27ac sites, H3K4me3 sites and 3 kb windows. The rates from these genomic regions were filtered to retain only those detected in greater than 10% of cells. The sPLS model was implemented assessing two components, selecting 50 features per component and per block (genomic region) in the DNA methylation and DNA accessibility datasets, and 100 genes per component in the transcriptome dataset. Further details are provided on GitHub (See ‘Data Availability’ below).

Heatmap visualization and identification of cell and expression clusters from sPLS-selected features was performed using the ComplexHeatmap package (v2.12.1). sPLS selected features for components 1 and 2 were extracted using the function ‘mixOmics::selectVar()’. Heatmap visualization was performed on sPLS-selected features using transcriptomic (converted to z-score) and epigenetic rates (mean of features in genomic regions and converted to z-score) that were entered into the model and included both treated and untreated cells. K-means clustering was performed on sPLS-selected transcriptomic features, first on Gene Expression (row_km=3), followed by Cell Group (column_km=4).

sPLS sample projections (Fig. 1D, F, and G) were plotted using ggplot2 (17) (v3.3.6) by extracting the sPLS components 1 and 2 for a given block (RNA or epigenetic genomic region) and overlaid with relevant information, that is, the cell group identified from k-means clustering and treated cell type or average DNA methylation.

Gene Ontology (GO) Over Representation Analysis (ORA) was calculated using clusterProfiler (18) (v4.4.4) for ‘biological process’ and displayed using enrichplot (v1.16.1) (Fig. 2H, I). Gene Expression k-means clusters (Fig. 1E) and sPLS selected features per component (1-2) were assessed by ‘enrichGO(p.adj=0.05, p.adj.method = "fdr", q.val.threshold = 0.4)’ with the list of genes from the batch corrected transcriptome dataset (entered into the sPLS model) as the background. Results were displayed as treeplots using default settings for pairwise ’termsim()’ and ‘treeplot(nCluster=5, showCategory = 10)’.

The correlation of sPLS features (Supplementary Fig. S5) was calculated as a similarity matrix using ‘mixOmics::circosPlot()’ on the sPLS model. The results were displayed using ComplexHeatmap, showing DNA methylation and DNA accessibility features related to transcript features split by the previously identified Gene Expression k-means clusters.

#### Locus-specific correlation analysis

To compare gene expression to adjacent epigenetic features, locus-specific correlations were performed using HL-60 scNMT-seq data (Fig. 1J). DNA methylation and DNA accessibility measurements were paired to genes based on annotation (promoters) or by strand-aware position within 10 kb of the gene transcription start site (CpG islands, H3K27ac sites, H3K4me3 sites and 3 kb windows). For paired sites, Pearson’s correlations were computed between CpG or GpC methylation rate and log gene expression values. All cells with data (i.e., the UNT, DAC, and AZA groups) were combined in these correlations, and a minimum of 22 cells with paired data (i.e., both gene expression and DNA methylation/accessibility measurements) were required for the correlation to be performed.

### Colony sequencing analyses

#### Colony TEM-seq and RNA-seq library preparation and sequencing

Single-colony TEM-seq (Fig. 2A, B, E) and parallel RNA-seq analyses (Fig. 2D, E and F) were performed as described (2, 5) with minor modifications. Lysates from single colonies (HL-60: 7.5 µL; MOLM-13 and MV-4-11: 2.5 µL) were used to separate genomic DNA and mRNA. During single-colony TEM-seq library preparation, the number of SINE Alu amplification cycles was reduced to 29. For RNA-seq analysis, 15 cycles of cDNA amplification were performed.

TEM-seq analysis of pooled colonies (Fig. 2C, Supplementary Fig. S12C) was performed as described (5) using 5-10 µL of cell lysate as input for bisulfite conversion and 29 cycles for SINE Alu amplification.

TEM-seq libraries were sequenced using 150 bp paired-end sequencing on the MiSeq platform. For RNA-seq libraries, paired-end 75 bp sequencing was performed on the NextSeq or NovaSeq platform.

#### TEM-seq data processing

Bisulfite sequencing data processing and extraction of global methylation estimates was performed as described above for single-cell scTEM-seq libraries.

#### RNA-seq data processing

Data processing was performed as described above for single-cell scTEM-seq libraries.

#### RNA-seq quality control, normalization and batch correction

Samples were excluded if they had less than 35% genes measured, or less than 70% reads in exons for HL-60 and MOLM-13 samples, or less than 65% reads in exons for MV-4-11 samples. RNA-seq data from single colonies were filtered to remove lowly expressed genes, requiring at least five counts in three samples. For each cell line, normalization was performed by ‘scuttle::logNormCounts()’ (v1.6.2) and batch corrected using mutual nearest neighbors method by ‘batchelor::mnnCorrect()’ (v1.12.3) with default parameters. Downstream analyses only considered autosomal genes (Chr1-22).

#### Highly variable gene analysis

For Figure 2D and E, highly variable genes (HVGs) were identified from colony RNA-seq data and principal component analysis (PCA) was performed using ‘scater::calculatePCA(ntop = 2,000)’. K-means clusters of HVGs were determined using the R stats package (v4.2.1) with ‘kmeans(centers = 8, iter.max = kmeans.iter, nstart = 50)’. Heatmapping of HVGs and k-means cluster was performed using ‘ComplexHeatmap::pheatmap()’ with z-scored values and Euclidean distance hierarchical clustering within row clusters (k-means groups) and columns (samples) ordered by treatment and descending average global methylation level.

GO ORA of k-means clusters was compared using clusterProfiler for ‘biological process’ by ‘compareCluster(pAdjustMethod = “fdr”, p.adj.threshold = 0.05, qvalueCutoff=0.4)’ and the full list of genes from the batch corrected dataset (for each cell type) as the background list. Plots were created using ‘clusterProfiler::dotplot(showCategory = 3) + coord_flip()’.

#### Correlation analysis in single-colony data

Pearson correlations comparing gene expression to mean global methylation in DAC HL-60 colonies (Supplementary Fig. S8) were performed using ‘cor.test()’ and underwent Benjamini–Hochberg false discovery rate adjustment using ‘p.adjust(method=”BH”)’. Gene clustering and heatmap visualization were performed for significantly correlated genes (p.adj ≤ 0.05 & cor.value.estimate ≤ -0.4 or cor.value.estimate ≥ 0.4). The average expression of each gene was calculated for each treatment group, with DAC split into high (≥75%) and low (<75%) global methylation groups. The R package pheatmap (v1.0.12) was used to plot the mean centered treatment group average expression levels with rows aggregated into 4 ‘kmeans_k’ clusters. The genes from each ‘Kmeans_k’ cluster were extracted and underwent GO ORA for biological process individually using 'enrichGO()’ with fdr adjustment, and the results are displayed as tree plots.

#### Analysis of cholesterol biosynthesis gene expression

For Supplementary Figure S11, genes from the cholesterol biosynthesis pathway (GO:0006695) were analyzed from batch corrected expression data for each cell line (HL-60, MOLM-13 and MV-4-11). After batch correction, 40 genes out of 58 genes (from GO:0006695) were in common across all three cell lines datasets. In total, for each cell line there were 41 (HL-60), 41 (MOLM-13) and 43 (MV-4-11) genes analyzed from GO:0006695. The R package rstatix (v0.7.2) was used to perform pairwise Wilcoxon test between treatment groups with UNT samples as the reference group for each gene. Benjamini-Hochberg false discovery rate (FDR) correction was performed on all p-values, per cell line. Boxplots were generated using ggpubr (v0.6.0) displaying the batch corrected relative expression with ‘pointrange’ error.

### Bulk RNA library preparation and sequencing

HL-60 cells were treated every 24 h for 72 h with HMAs (100nM DAC, 2000nM AZA). Cells (1x10^6^) were stored in 1x DNA/RNA Shield (Zymo Research) and RNA was extracted using the Quick DNA/RNA MagBind kit (Zymo Research), according to manufacturer’s instructions. RNA was quantified on a Nanodrop, and integrity was assessed using a TapeStation RNA ScreenTape. RNA-seq libraries (700ng RNA input) were prepared using the TruSeq Stranded mRNA kit (Illumina), according to the manufacturer’s instructions, and sequenced on the NovaSeq 6000 system (SP flow cell; 150 cycles). Sequencing data was analysed using Seqmonk (QC and DESeq2).

### Post-bisulphite adaptor tagging (PBAT) library preparation and sequencing

DNA methylation analysis presented in Supplementary Fig. S1 was performed using a post-bisulphite adaptor tagging (PBAT) sequencing approach as previously described (2).

### Bulk TEM-seq library preparation and sequencing

For analysis in Supplementary Fig. S2, CellTrace-low (bottom 30-35%), -high (top 15-20%) and -medium (middle 40-50%) populations were purified by FACS and resuspended in 30 µL RLT PLUS buffer (Qiagen) containing 30 U SUPERas-In (Thermo Fisher Scientific). Lysates were stored at -80 °C for TEM-seq analysis.

TEM-seq analysis of CellTrace-low, -medium and -high populations was performed as described (2, 5) using 5-10 µL of lysate as input for bisulfite conversion and 25 cycles for SINE Alu amplification.

### AML xenograft models

Five-week-old female NOD.Cg-Prkdc scid Il2rg tm1Wjl /SzJ (NSG) mice were obtained from the Australian Bioresources (ABR, Moss Vale, NSW, Australia) and acclimatized for one week prior to any experimental procedure.

MOLM-13 cells transduced with firefly luciferase (MOLM-13-luc; 5x10^5^ cells suspended in 100 μL of PBS) were injected into the lateral tail vein of NSG mice. Leukemia burden was assessed by bioluminescence imaging (BLI) twice a week using an IVIS Spectrum *in vivo* imaging system (PerkinElmer, Waltham, MA, USA), following intraperitoneal injections of 150 mg/kg D-luciferin (Promega, Alexandria, NSW, Australia) under anesthesia with isoflurane. Treatments commenced on day 6 post-engraftment when a positive luminescence signal was detected. Mice (*n* = 5-15/group) were treated by intraperitoneal injection (IP) of either vehicle (2% DMSO, 30% PEG300 in water), DAC (0.2 mg/kg in saline), rosuvastatin (1 mg/kg), or combination rosuvastatin (1 mg/kg, 10 mg/kg, or 40 mg/kg in 30% PEG300 in water) and DAC (0.2 mg/kg) once a day (5 days on, 2 days off) for up to 3 weeks. The animals were monitored until they reached the ethical endpoint.

For patient derived xenograft (PDX) experiments, NSG mice were inoculated with 2x10^6^ AML-16 cells (*FLT3-ITD*, *NPM1*, *IDH2*, and *WT1* mutant) (19) in 100 µL of PBS, by injection into the lateral tail vein. Leukemia burden (% hCD45+ cells in peripheral blood) was assessed 1-2 times weekly via flow cytometry (FACSCanto II). Treatment commenced once an average leukemia burden of 1% CD45^+^ cells was achieved (~3.5 weeks). Mice (n= 6-11 per group) received vehicle (2% DMSO, 30% PEG300 in water), DAC (0.2 mg/kg), AZA (1 mg/kg), rosuvastatin (1 mg/kg), DAC + rosuvastatin, or AZA + rosuvastatin, via IP injections once a day (5 days on, 2 days off) for 1 week (cycle 1), followed by twice per week (dispersed) for an additional 2 weeks (2 cycles). Mice were monitored until the ethical endpoint (>25% huCD45^+^ cells).

#### Survival Analysis

Survival analyses were performed using Kaplan-Meier analysis followed by the log-rank (Mantel-Cox) test. All statistical analyses were performed using GraphPad Prism v. 9.0 (GraphPad Software, La Jolla, CA, USA). Differences were considered statistically significant at a *p*-value < 0.05.

### Consideration of ARRIVE guidelines relating to animal experiments

#### Animal housing

NSG mice were kept at the Bioresources Facility (Callaghan, NSW, Australia) at 22 ± 2°C, with water and chow *ad libitum*, under a 12:12 h light and dark photoperiod, and housed in ventilated cages in a pathogen-free environment.

#### Group size

G*Power 3.1 (20) was used to perform calculations on sample size, effect size, and statistical power. The minimal significance (α) and statistical power (1-β) were set at 0.05 and 0.80, respectively. Calculations were carried out for two groups by using Student’s t-distribution.

#### Randomisation and blinding

For the cell line-derived xenograft model (MOLM-13-*luc)*, once the luminescence signal was detected (day 6 post-engraftment), mice were randomly allocated to treatment groups. The average BLI of each group was calculated to ensure that tumour burden was equivalent between treatments. Mice with low BLI (negative signal at day 6 post-engraftment) were excluded from the study. For the patient-derived xenograft model (AML-16), once around 1% of leukemia burden was detected in the blood of mice (~3.5 weeks post-engraftment), mice were randomly allocated to treatment groups. The average % of leukemia burden of each group was calculated to ensure that burden was equivalent between treatment groups.

#### Animal monitoring

Following xenotransplantation, animals were monitored daily for clinical and behavioural changes, and body weight was recorded twice a week with a digital balance. Ethical endpoint was defined according to a monitoring checklist (inhibited activity, lethargy, moribund animal, loss of ≥20% of initial body weight, ≥25% leukemia burden – for AML-16 model). All animals were euthanised by carbon dioxide asphyxiation at ethical endpoint.

### Supplementary References

1. Clark SJ, Argelaguet R, Kapourani CA, Stubbs TM, Lee HJ, Alda-Catalinas C, et al. scNMT-seq enables joint profiling of chromatin accessibility DNA methylation and transcription in single cells. Nature Communications. 2018;9(1):781.

2. Hunt KV, Burnard SM, Roper EA, Bond DR, Dun MD, Verrills NM, et al. scTEM-seq: Single-cell analysis of transposable element methylation to link global epigenetic heterogeneity with transcriptional programs. Sci Rep. 2022;12(1):5776.

3. Krueger F, Andrews SR. Bismark: a flexible aligner and methylation caller for Bisulfite-Seq applications. Bioinformatics. 2011;27(11):1571-2.

4. F K. TrimGalore. 0.6.10 ed: <https://github.com/FelixKrueger/TrimGalore> 10.5281/zenodo.7598955; 2023.

5. Hunt KV BS, Bond DR, Lee HJ. Protocol for targeted analysis of transposable element methylation levels and transcriptome in single cells using scTEM-seq. ProtocolExchange. 2022.

6. Kim D, Paggi JM, Park C, Bennett C, Salzberg SL. Graph-based genome alignment and genotyping with HISAT2 and HISAT-genotype. Nature Biotechnology. 2019;37(8):907-15.

7. Jin Y, Tam OH, Paniagua E, Hammell M. TEtranscripts: a package for including transposable elements in differential expression analysis of RNA-seq datasets. Bioinformatics. 2015;31(22):3593-9.

8. An integrated encyclopedia of DNA elements in the human genome. Nature. 2012;489(7414):57-74.

9. Smallwood SA, Lee HJ, Angermueller C, Krueger F, Saadeh H, Peat J, et al. Single-cell genome-wide bisulfite sequencing for assessing epigenetic heterogeneity. Nature Methods. 2014;11(8):817-20.

10. L. Lun AT, Bach K, Marioni JC. Pooling across cells to normalize single-cell RNA sequencing data with many zero counts. Genome Biology. 2016;17(1):75.

11. McCarthy DJ, Campbell KR, Lun ATL, Wills QF. Scater: pre-processing, quality control, normalization and visualization of single-cell RNA-seq data in R. Bioinformatics. 2017;33(8):1179-86.

12. Haghverdi L, Lun ATL, Morgan MD, Marioni JC. Batch effects in single-cell RNA-sequencing data are corrected by matching mutual nearest neighbors. Nature Biotechnology. 2018;36(5):421-7.

13. Hafemeister C, Satija R. Normalization and variance stabilization of single-cell RNA-seq data using regularized negative binomial regression. Genome Biology. 2019;20(1):296.

14. Hao Y, Hao S, Andersen-Nissen E, Mauck WM, 3rd, Zheng S, Butler A, et al. Integrated analysis of multimodal single-cell data. Cell. 2021;184(13):3573-87.e29.

15. Rohart F, Gautier B, Singh A, Le Cao KA. mixOmics: An R package for 'omics feature selection and multiple data integration. PLoS Comput Biol. 2017;13(11):e1005752.

16. Rohart F, Eslami A, Matigian N, Bougeard S, Lê Cao K-A. MINT: a multivariate integrative method to identify reproducible molecular signatures across independent experiments and platforms. BMC Bioinformatics. 2017;18(1):128.

17. Wickham H. ggplot2: Elegant Graphics for Data Analysis: Springer-Verlag New York; 2016.

18. Wu T, Hu E, Xu S, Chen M, Guo P, Dai Z, et al. clusterProfiler 4.0: A universal enrichment tool for interpreting omics data. The Innovation. 2021;2(3):100141.

19. Lee EM, Yee D, Busfield SJ, McManus JF, Cummings N, Vairo G, et al. Efficacy of an Fc-modified anti-CD123 antibody (CSL362) combined with chemotherapy in xenograft models of acute myelogenous leukemia in immunodeficient mice. Haematologica. 2015;100(7):914-26.

20. Faul F, Erdfelder E, Buchner A, Lang AG. Statistical power analyses using G*Power 3.1: tests for correlation and regression analyses. Behav Res Methods. 2009;41(4):1149-60.

## Supplementary Figures

**Fig. S1: Low-dose HMA treatment reduces DNA methylation without acute cytotoxicity in AML cell lines.** AML cell lines (HL-60, MOLM-13, and MV-4-11) were treated every 24 h for a total of 72 h, with various doses of decitabine (DAC: 10, 100, 500nM) and azacytidine (AZA: 100, 500, 2000nM), or untreated (UNT) in suspension culture. **A)** Cell growth and viability was measured throughout the dose response experiment using trypan blue exclusion assay (viable cells/mL and % viable cells). **B)** Following 72 h HMA treatment, average DNA methylation levels (%) were assessed using Post-bisulphite Adapter Tagging (PBAT). Time-course experiment showing: **C)** cell viability (total viable cells, left; % viable cells, right), **D)** cell division (CellTrace fluorescence), and **E)** average methylation (PBAT) at various time points following treatment with 100nM DAC and 2000nM AZA in HL-60 cells. All data shown as mean +/- SEM, *n=3* experiments. Statistical analysis (A) was performed using two-way repeated measures (RM) ANOVA with Dunnett's multiple comparisons test (p<0.04*, p<0.002**), compared to respective UNT within each time point. Statistical analysis (B) was performed using ordinary one-way ANOVA with Dunnett's multiple comparisons test (*p* < 0.03*), compared to UNT. Statistical analysis (C - E) was performed using two-way repeated measures (RM) ANOVA with Dunnett's multiple comparisons test (*p* < 0.04*, *p* < 0.008**), compared to respective UNT within each time point.


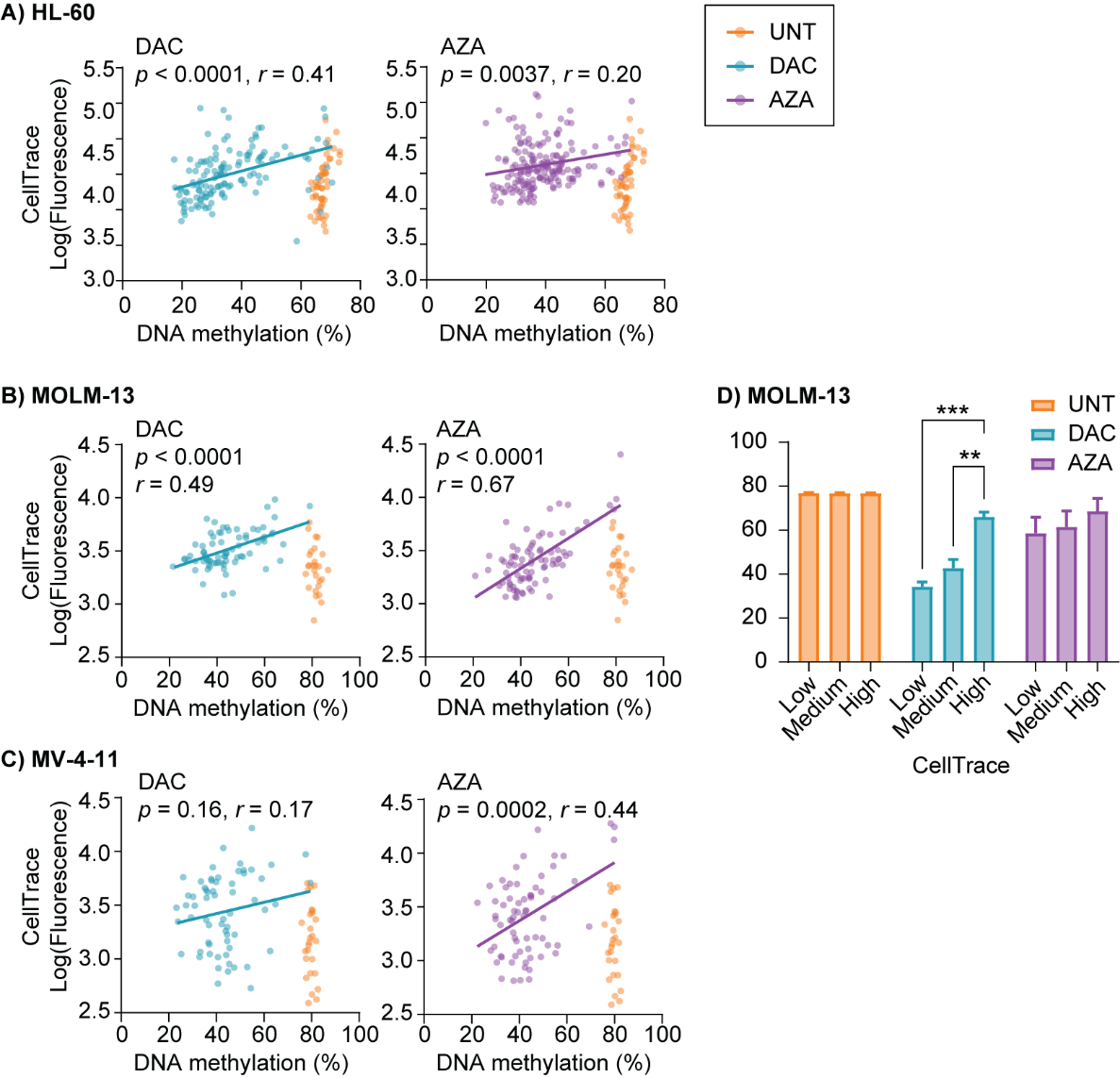


**Fig. S2: Slowly dividing AML cells retain high levels of DNA methylation during HMA treatment.** HL-60, MOLM-13 and MV-4-11 cells were labelled with CellTrace and treated with decitabine (DAC; 100nM) or azacytidine (AZA; HL-60: 2000nM, MOLM-13 and MV-4-11: 500nM) every 24 h for 72 h. **A - C)** Data from Figure 1C are replotted with the addition of untreated cells from Figure 1A (UNT, orange), and an additional 138 scBS-seq libraries from HL-60 cells (UNT = 16, DAC n=38, AZA n = 84). Linear regressions for DAC (cyan, left) and AZA (purple, right) groups are shown with F-test *p*-values, and Pearson correlation coefficients (*r*). **D)** Populations of CellTrace-high, -medium, and -low MOLM-13 cells were collected by FACS and TEM-seq analysis of DNA methylation at SINE Alu sites was performed. Data are shown as mean and range for duplicate experiments. Statistical analysis was performed using ordinary two-way ANOVA with Dunnett’s multiple comparisons test: * < *p* < 0.05, ** *p* < 0.01, *** *p* = 0.001 vs. CellTrace-high.

**
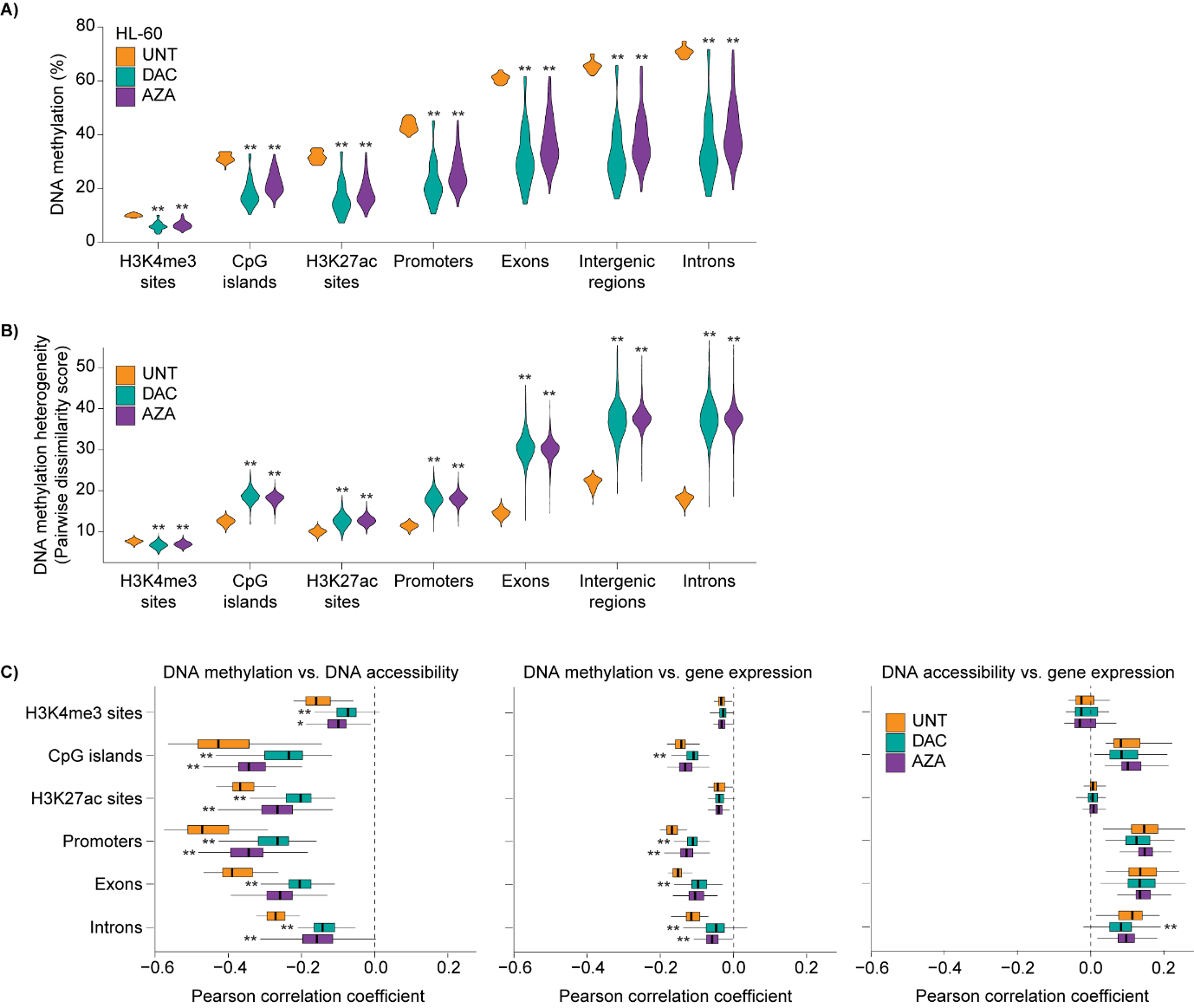
**

**Fig. S3: HMA-induced DNA methylation heterogeneity is observed in most genomic contexts. A)** Violin plots of absolute DNA methylation levels in different genomic contexts from HL-60 scNMT-seq data. **B)** Violin plots of DNA methylation heterogeneity, as determined by pairwise dissimilarity analysis, within different genomic contexts from HL-60 scNMT-seq data. **C)** Box and whisker plots of Pearson correlation coefficients computed within single cells from HL-60 scNMT-seq data. DNA methylation and DNA accessibility were considered in different genomic contexts, and individual loci were matched based on genomic co-ordinates. Correlations were performed between DNA methylation and DNA accessibility (left), DNA methylation and gene expression (middle), and DNA accessibility and gene expression (right). Boxes depict the interquartile range (IQR) with median. Whiskers extend to the highest and lowest data points within 1.5 x IQR of the first and third quartile. Outlying data points are not shown. Statistical analysis was performed using ordinary one-way ANOVA with Dunnett’s (A) or Šídák’s (C-E) multiple comparisons test: * *p* < 0.05, ** *p* < 0.0005 vs. UNT.


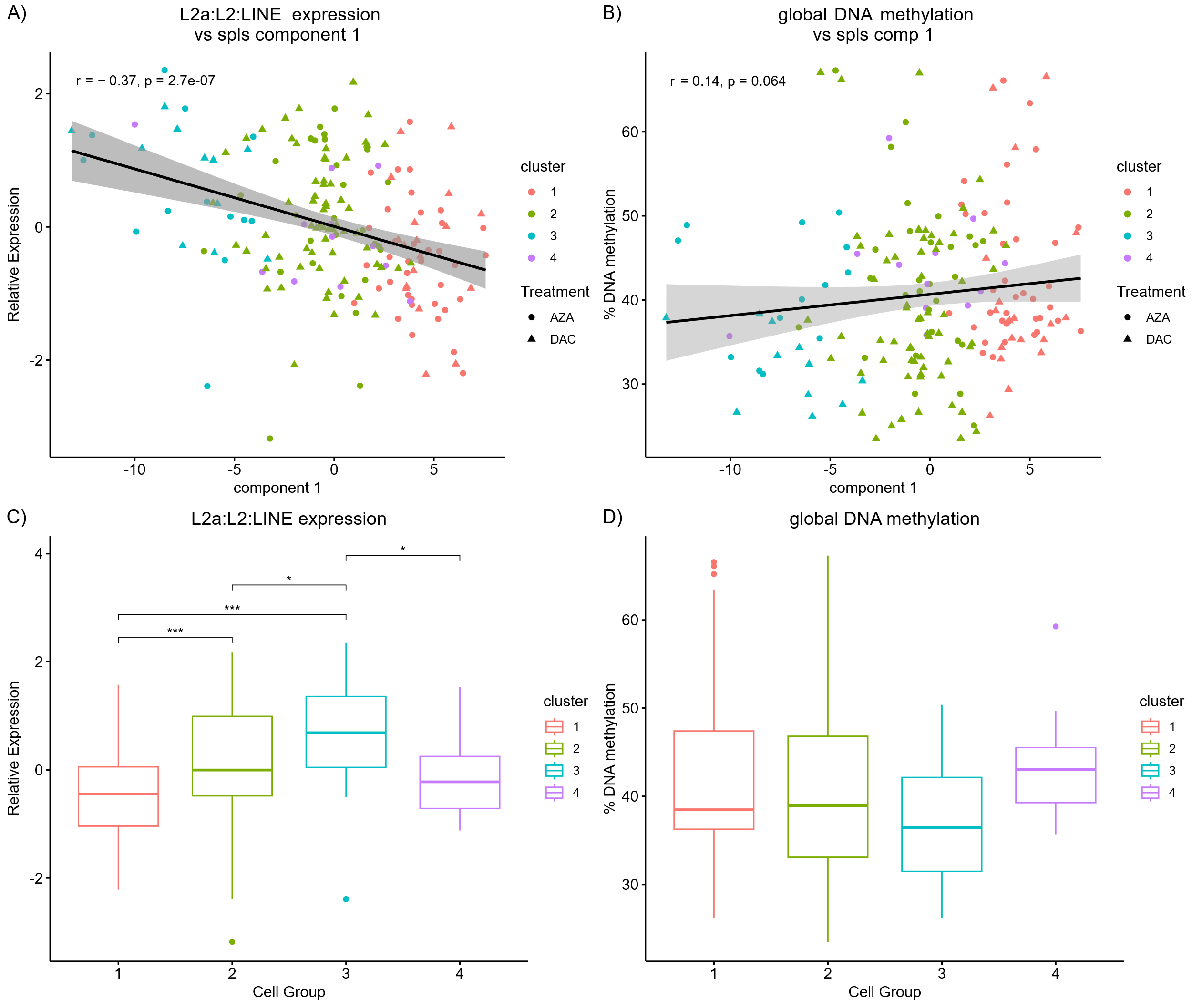


**Fig. S4**: **LINE:L2a expression and DNA methylation correlations with sPLS component 1 and comparisons between cell groups.** **A)** Expression of L2a and **B)** global DNA methylation plotted against sPLS component 1 values for HMA treated cells. Fitted linear models and statistical testing by spearman’s rank correlation are included. **C)** L2a expression and **D)** global DNA methylation are compared between cell groups identified from sPLS analysis (Fig. 1E). Boxes depict the interquartile range (IQR) with median. Whiskers extend to the highest and lowest data points within 1.5 x IQR of the first and third quartile. Significance was determined by Kruskal Wallis testing and post-hoc analysis by pair-wise Wilcoxon rank sum testing with Benjamini-Hochberg correction: * p ≤ 0.05, ** p ≤ 0.01, ***, p ≤ 0.001.


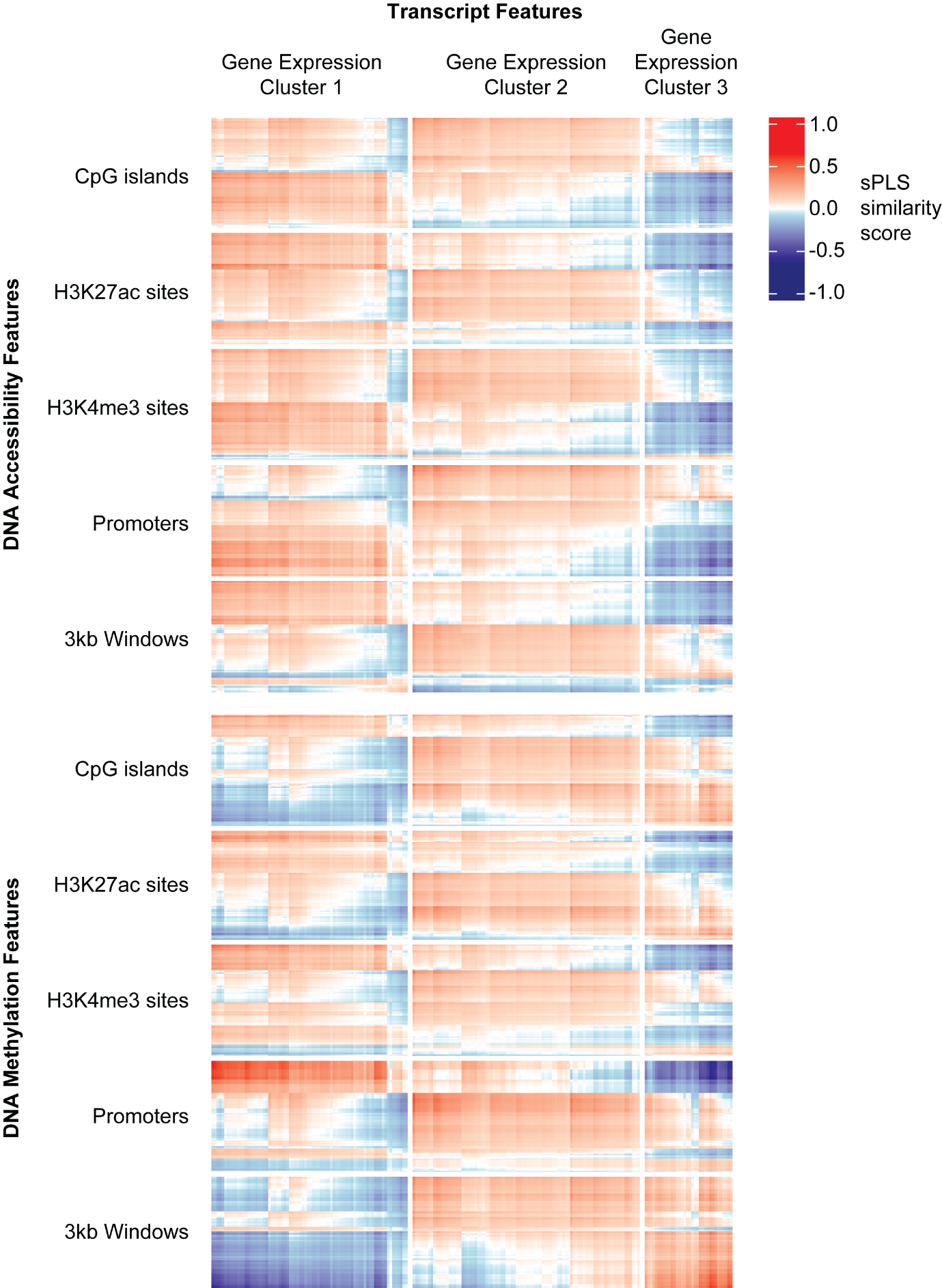


**Fig. S5: Transcript and epigenetic features selected by sPLS**. Similarity score heatmap computed by mixomics::circosPlot() using sPLS results shown in Figure 1E, and supplementary tables 3, 5 and 6.

**
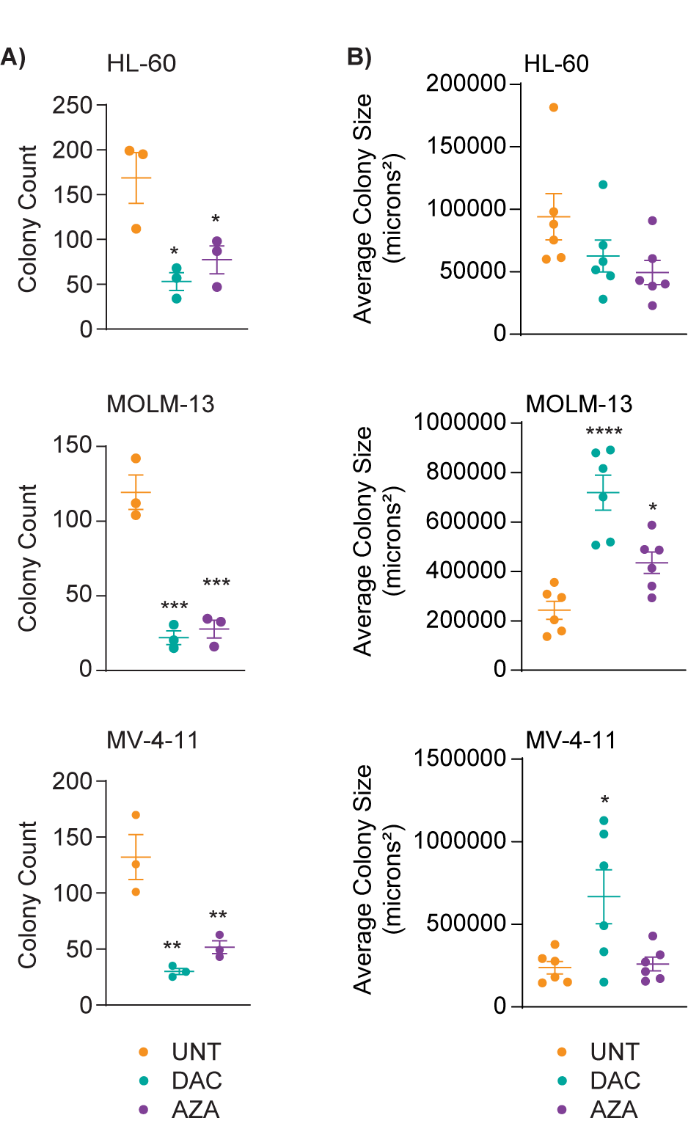
**

**Fig. S6: HMA treatment influences AML cell self-renewal.** AML cell lines were treated with DAC or AZA in suspension culture as described in Figure 1. On experiment day 3, cells were seeded in MethoCult media for colony formation, without additional HMA treatment. **A)** Colony counts performed on experiment day 17 for HL-60, MOLM-13, and MV-4-11 cells after treatment with DAC (cyan) or AZA (purple) vs. untreated cells (UNT, orange). Statistical analysis was performed using ordinary one-way ANOVA with Dunnett’s multiple comparisons test with a *p* < 0.05 cut-off for significance (*p* < 0.03*, *p* < 0.006**, *p* < 0.0002***). **B)** Average size of colonies (microns^2^) formed following treatment with DAC (cyan) or AZA (purple) for 72 hours prior to colony-forming assays. Data are expressed as mean +/- standard error of the mean (SEM). Statistical analysis using Ordinary one-way ANOVA with Dunnett's multiple comparisons test (*p* < 0.0001****, *p* < 0.03*) compared to UNT. Experiments were performed in biological triplicate (*n=3*), with technical duplicates.

**
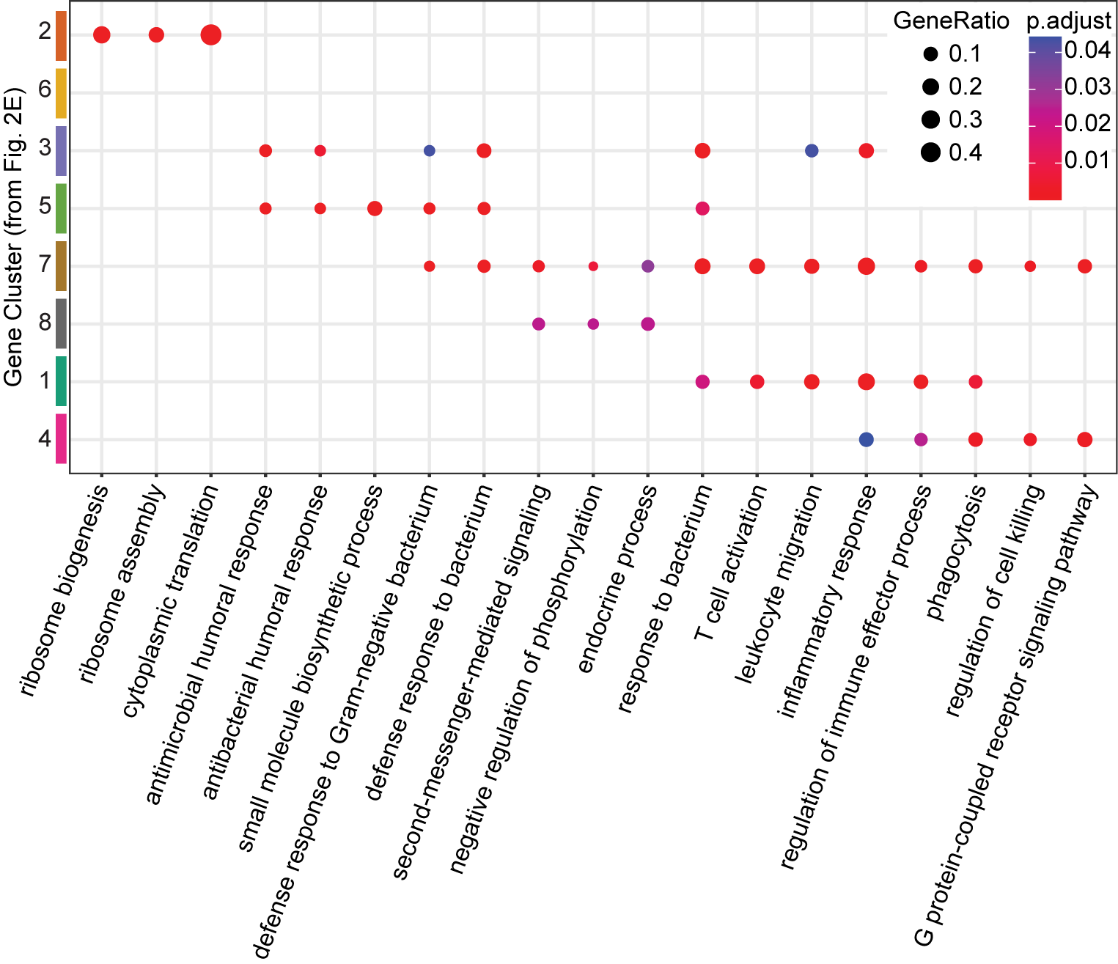
**

**Fig. S7: Over-represented gene ontology (GO) terms among highly-variable genes in single-colony RNA-seq data.** GO analysis of the clusters from the top 2000 highly variable genes from Figure 2E. The size of the circles indicates the gene ratio (number of genes from the input list annotated to the GO term divided by the total number of genes in the input list), and the color represents the significance of the adjusted *p*-value. Gene clusters are color-coded on the y-axis and GO processes are shown on the x-axis.


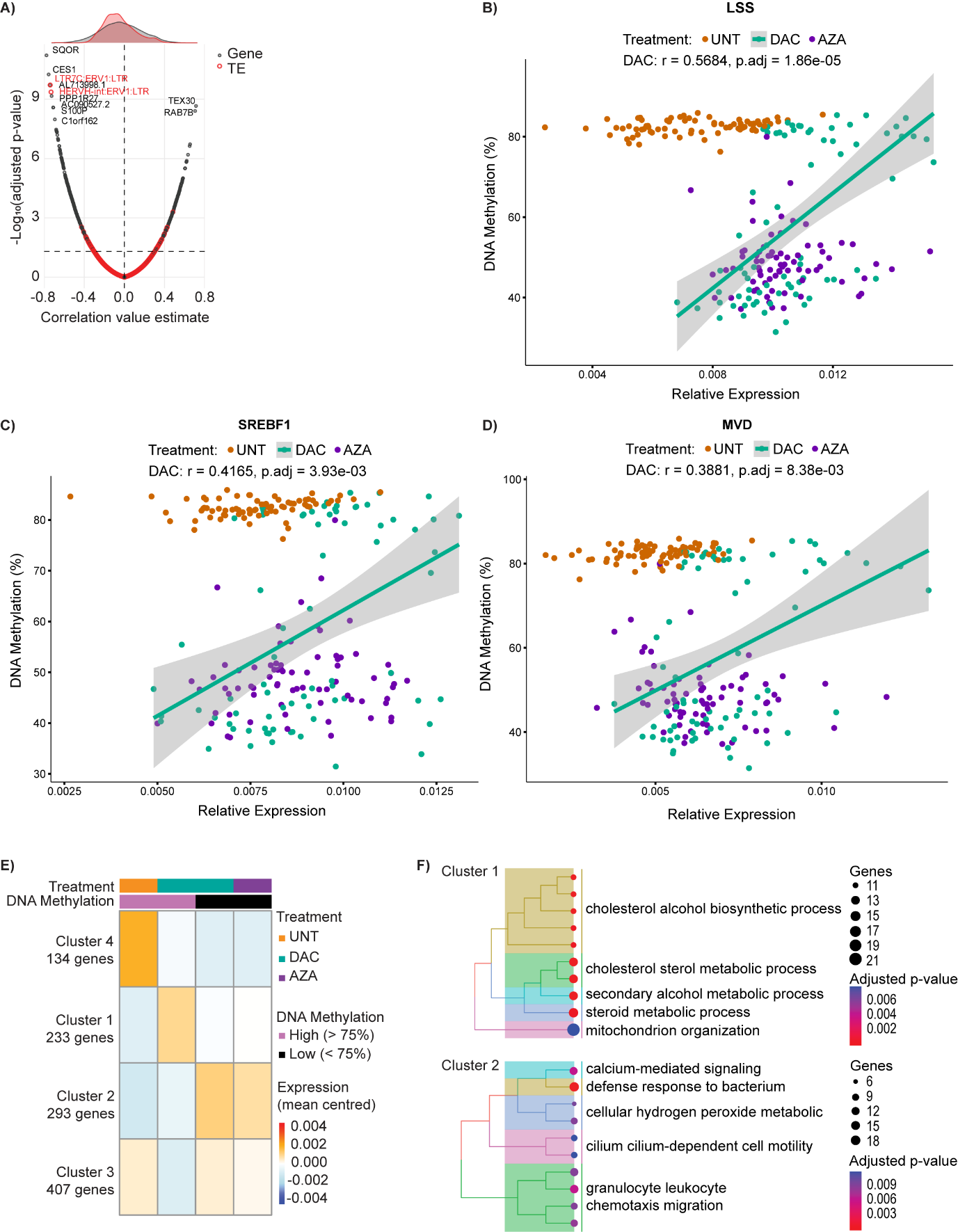


**Fig. S8: Correlations between gene expression and global DNA methylation in HL-60 colonies formed after decitabine (DAC) treatment. A)** Volcano plot showing the Pearson correlation coefficient and adjusted *p*-value for correlations between gene or transposable element (TE) expression and global DNA methylation levels from HL-60 colonies derived following DAC treatment. The upper density plot shows a bias toward negative correlations, especially between global DNA methylation level and TE expression (red). **B) to D)** Scatter plots display representative correlations for three genes from ‘cholesterol biosynthetic process’ (GO:0006695): *LSS* (B), *SREBF1* (C), *MVD* (D). Linear model regression lines for DAC-treated cells were plotted by R displaying standard error intervals. Correlation coefficients (r) and adjusted p-values are from the correlation analysis in Table S10. Data from untreated (UNT) and azacytidine (AZA) samples are also shown. **E)** Simplified heatmap of k-means clustering for the 1,067 genes with significant correlations to global DNA methylation level (adjusted *p*-value ≤ 0.05 and 0.4 ≤ correlation estimate ≤ 0.4, from A). **F)** Summarised tree plots displaying GO terms with significant (adjusted *p*-value < 0.05) over-representation in clusters 1 and 2 (from E).

**
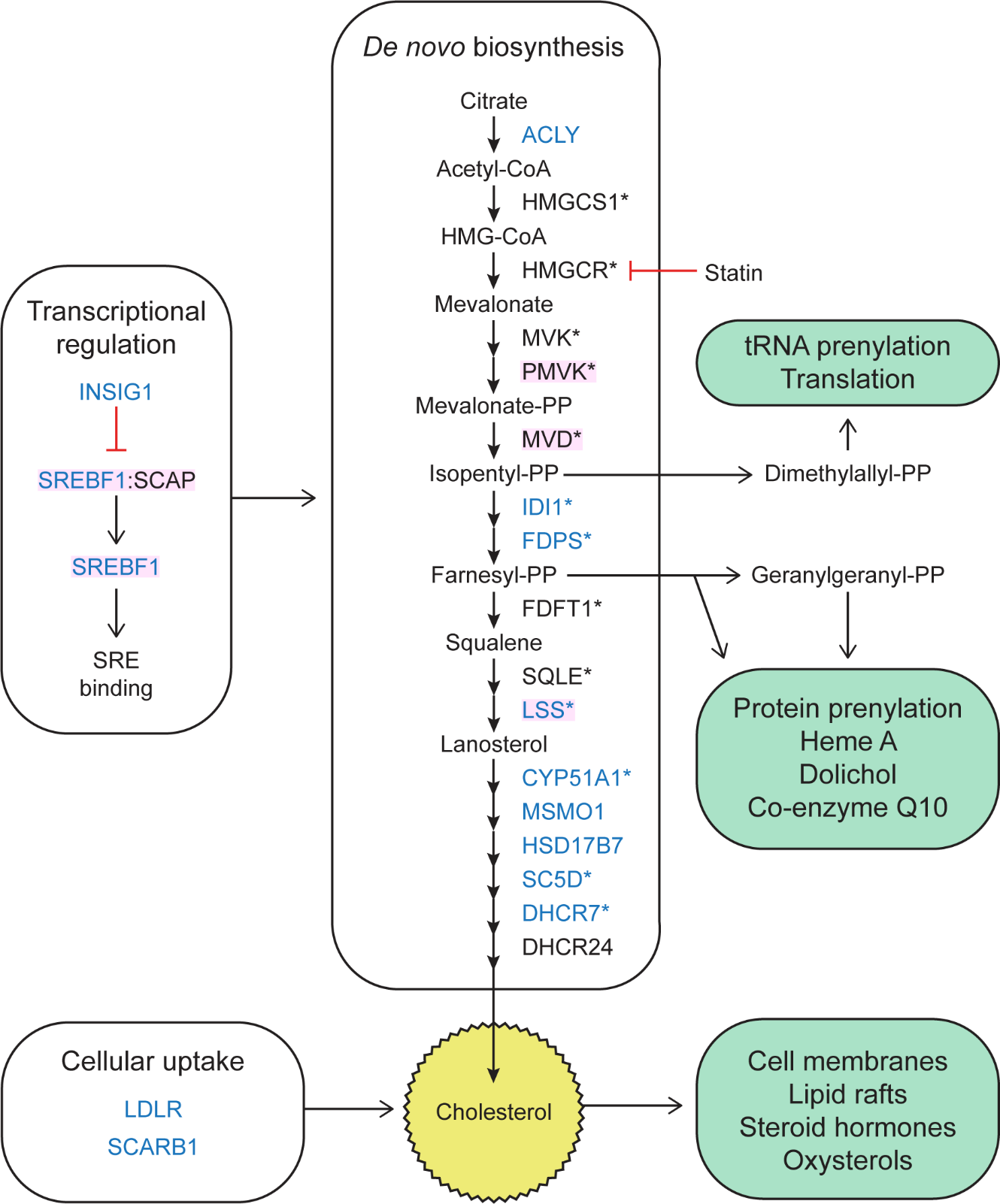
­­**

**Fig. S9: Genes involved in cholesterol biosynthesis.** Schematic illustration showing the contribution of selected genes to cholesterol regulation. Pink shading indicates genes that were significantly increased (Pair-wise Wilcoxon test with Benjamini-Hochberg correction) in all cell lines by both DAC and AZA (Fig. S11). Blue symbols indicate genes with positive correlations between expression and global DNA methylation among DAC-treated HL-60 colonies (cluster 1, Supplementary Fig. S8E). *SREBF1* encodes the SREBP1 transcription factor, which binds to sterol regulatory elements (SRE) to control the expression of target genes. Genes with promoter SREBP1 binding (according to Reactome pathway #R-HSA-2426168) are marked with an asterisk. Statins inhibit the HMGCR enzyme. Green boxes list downstream functions of cholesterol and other metabolites produced during cholesterol biosynthesis.

**
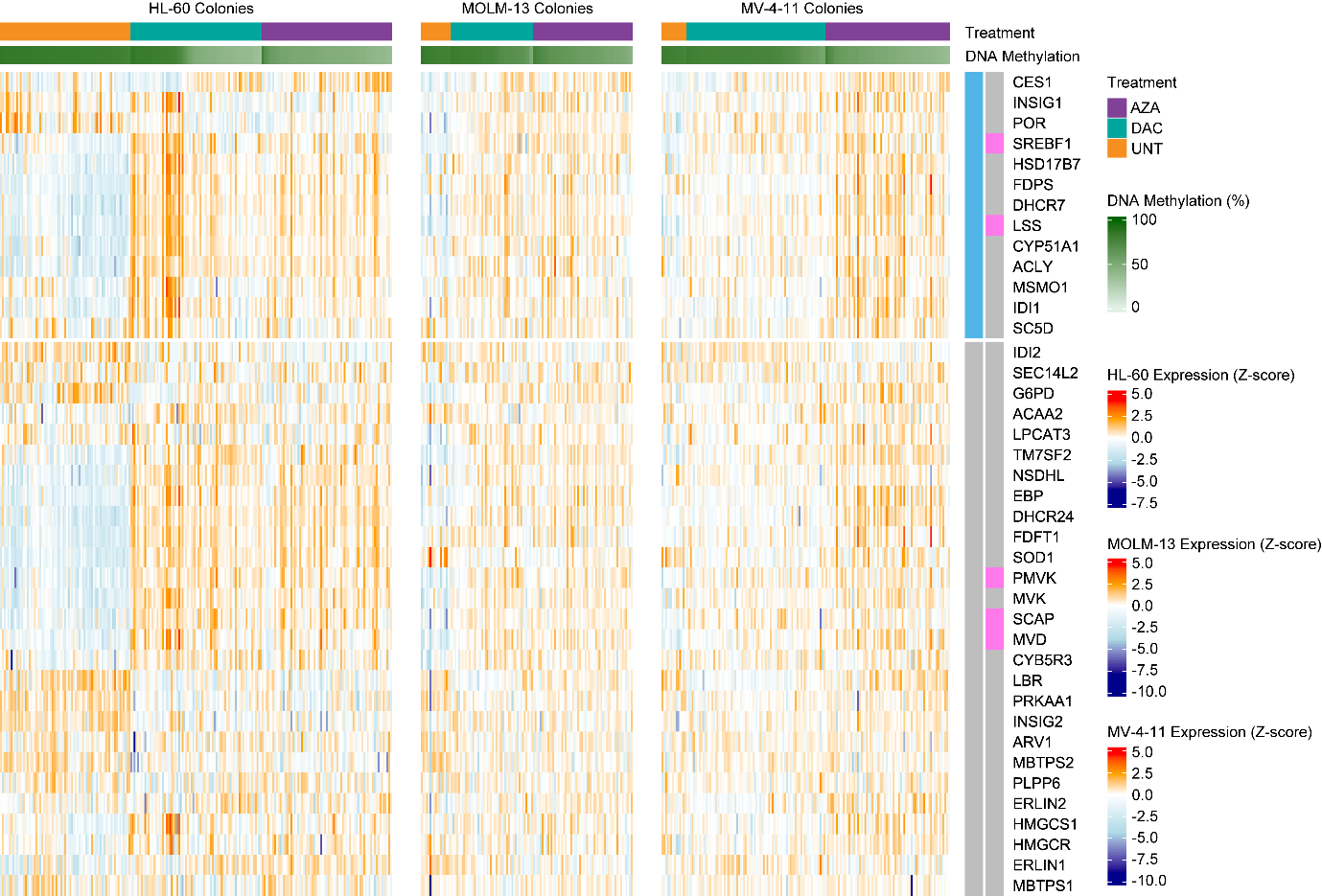
**

**Fig. S10: Expression of genes from ‘cholesterol biosynthesis process’ (GO:0006695) in colonies from all 3 cell lines.** Genes with positive correlations between expression and global DNA methylation among DAC-treated HL-60 colonies (cluster 1, Fig. S8E) are indicated by the blue side bar. Genes that were significantly increased in all cell lines by both DAC and AZA (Pair-wise Wilcoxon test with Benjamini-Hochberg correction, Fig. S11) are also indicated (pink side bar).


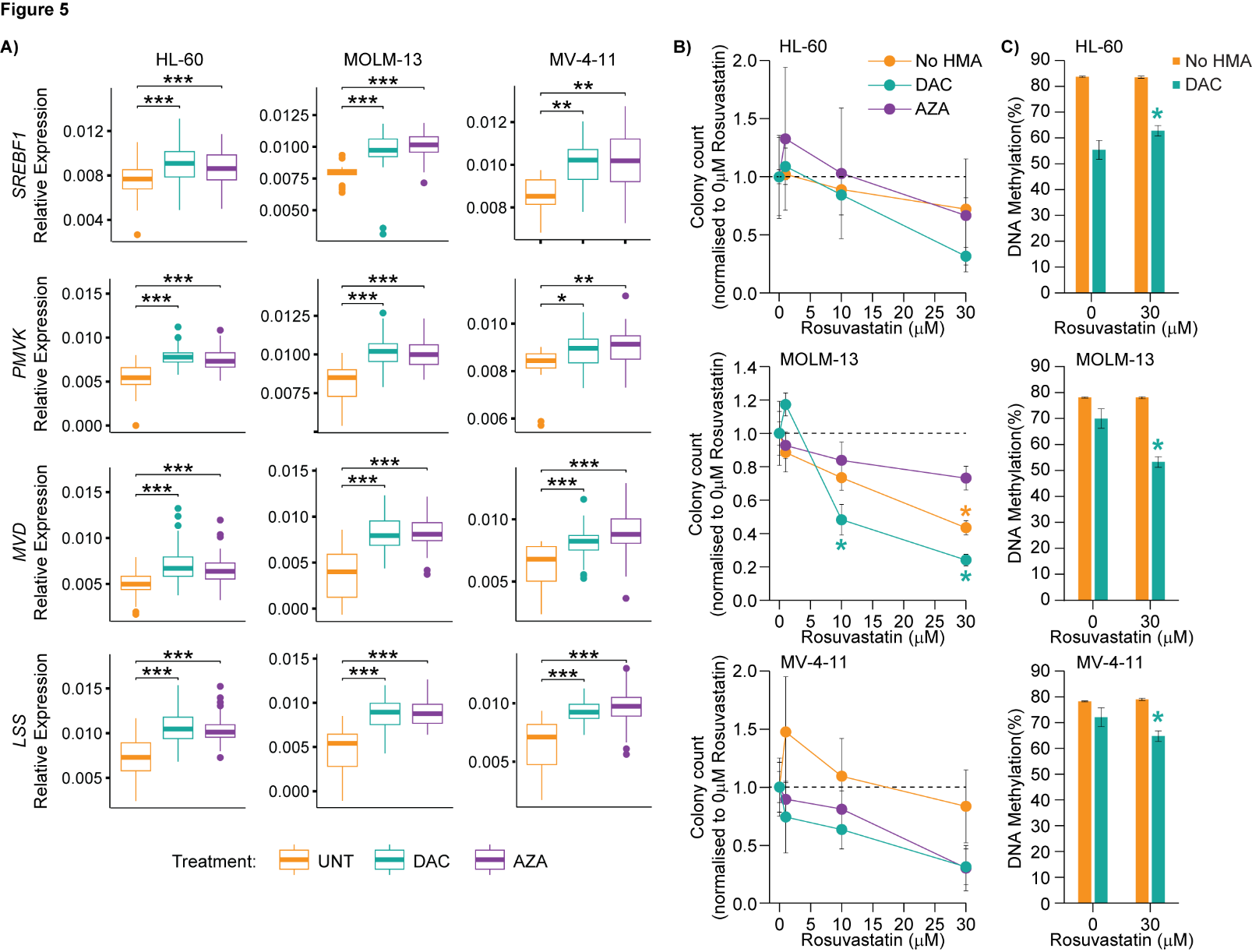


**Fig. S11: Expression of selected cholesterol-related genes in colonies from all 3 cell lines.** Box and whisker plots of relative expression for *SREBF1*, *PMVK*, *MVD* and *LSS* in HL-60 (left), MOLM-13 (middle) and MV-4-11 (right) colony data. Pairwise Wilcoxon test between treatment groups was performed with UNT samples as the reference group, for genes present within ‘cholesterol biosynthetic process’ (GO:0006695). Boxes depict the interquartile range (IQR) with median. Whiskers extend to the highest and lowest data points within 1.5 x IQR of the first and third quartile. Benjamini-Hochberg false discover rate (FDR) correction was performed on all p-values, per cell line: FDR < 0.05*, FDR < 0.01**, FDR < 0.001***.

**D)**


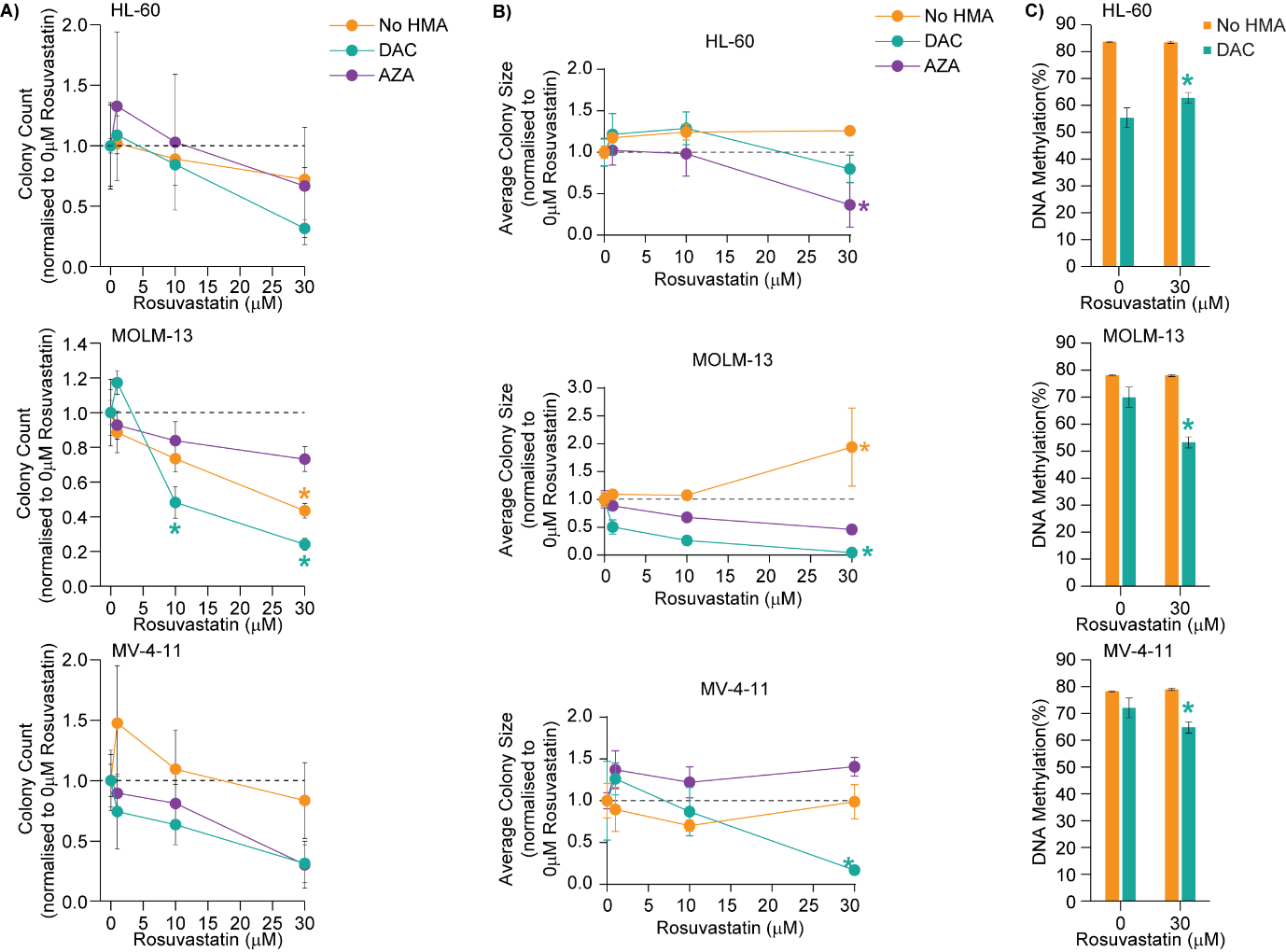


**Fig. S12: Rosuvastatin co-treatment enhances HMA effects in colony-forming assays.** AML cell lines were treated with DAC or AZA in suspension culture as in Figure 1. On experiment day 3, cells were washed and seeded in MethoCult media for colony formation, with addition of rosuvastatin (0, 1, 10, 30 µM; single dose). On experiment day 17, colonies were counted and collected for sequencing analysis. **A)** Colony counts for HL-60 (top), MOLM-13 (middle) and MV-4-11 (bottom) cell lines obtained following HMA and rosuvastatin co-treatments. Data from No HMA (orange), DAC (cyan) and AZA (purple) groups are normalised to the corresponding 0 μM rosuvastatin control. Means ± SEM for *n = 3* experiments. Significance determined by two-way ordinary ANOVA with Dunnett’s multiple comparisons test, *p* < 0.05* vs. corresponding 0 µM rosuvastatin control. **B)** Average size of colonies following treatment with no HMA, DAC or AZA and various doses of rosuvastatin (0, 1, 10, 30 µM). For each HMA treatment, colony sizes are normalised to the 0 µM rosuvastatin control, *n = 3*. Statistical analysis using Ordinary two-way ANOVA with Dunnett's multiple comparisons test (*p* < 0.04*) compared to corresponding 0 µM rosuvastatin control. All data is shown as mean +/- SEM. **C)** DNA methylation of day 17 colonies formed following DAC and rosuvastatin co-treatments. Means ± SEM for *n = 3* experiments. Significance determined by one-way ANOVA with Tukey’s multiple comparisons test, *p* < 0.05* vs. corresponding 0 µM rosuvastatin control. **D)** Colony counts from A were normalised to untreated colony counts and the coefficient of drug interaction (CDI) was calculated using the following formula: CDI = AB / (A x B). CDI values of 1, <1 or >1 express additive, synergistic or antagonistic effects, respectively. A CDI value < 0.7 demonstrates a strong synergism of the drug combination (HMA + ROS). DAC (100nM) combined with ROS (30 µM) showed synergism in all three AML cell lines with respect to inhibition of self-renewal capacity (colony formation), however, synergism was only seen in MV-4-11 cells when AZA (500nM) was combined with ROS (30 µM).

**
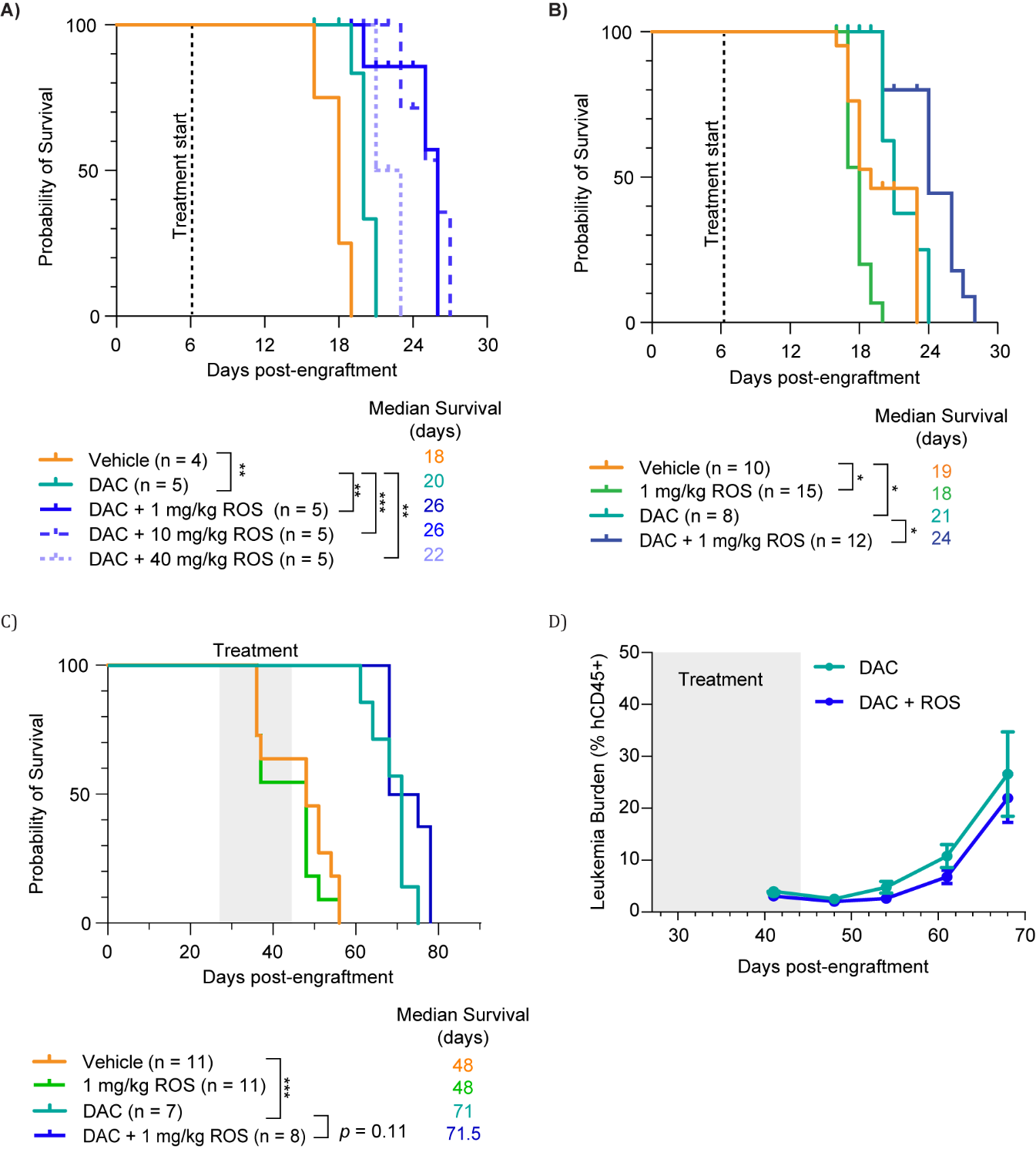
**

**Fig. S13: HMA and statin co-treatment increases survival *in vivo*.** **A)** Rosuvastatin dose optimisation experiment showing median survival of NSG mice engrafted with MOLM-13-*luc* cells following treatment with DAC (0.2 mg/kg/day) +/- rosuvastatin (1, 10, 40 mg/kg/day) on a treatment schedule of ‘5 days on, 2 days off’ for 3 cycles via intraperitoneal (IP) injection. **B)** Validation of survival benefit when DAC (0.2 mg/kg/day) is combined with rosuvastatin (1 mg/kg/day) in mice engrafted with MOLM-13-*luc* AML cells. **C)** Survival analysis for AML-16 patient-derived xenograft model following treatment with vehicle, rosuvastatin (1 mg/kg/day), DAC (0.2 mg/kg/day), or DAC (0.2 mg/kg/day) + rosuvastatin (1 mg/kg/day) on a treatment schedule of ‘5 days on, 2 days off’ for the first cycle, followed by two times (dispersed) per week for the remaining two cycles, via IP injection. Statistical analysis was performed using Kaplan-Meier analysis followed by the Log-rank (Mantel-Cox) test and a *p*-value of < 0.05 was considered statistically significant. **D)** Leukemia burden (% human CD45^+^ cells) for experiment presented in C. The Mann-Whitney test (unpaired, non-parametric, two-tailed t-test) was used to test for statistical significance with a *p*-value cut-off of 0.05.
